# Comprehensive analysis of the *Gossypium hirsutum* L. respiratory burst oxidase homolog (*Ghrboh*) gene family

**DOI:** 10.1186/s12864-020-6503-6

**Published:** 2020-01-29

**Authors:** Wei Wang, Dongdong Chen, Dan Liu, Yingying Cheng, Xiaopei Zhang, Lirong Song, Mengjiao Hu, Jie Dong, Fafu Shen

**Affiliations:** State Key Laboratory of Crop Biology, College of Agronomy, Shandong Agricultural University, NO. 61 Daizong Street, Tai’an, Shandong 271018 People’s Republic of China

**Keywords:** Rboh, Reactive oxygen species, Upland cotton, Expression patterns, Gene family

## Abstract

**Background:**

Plant NADPH oxidase (NOX), also known as respiratory burst oxidase homolog (rboh), encoded by the *rboh* gene, is a key enzyme in the reactive oxygen species (ROS) metabolic network. It catalyzes the formation of the superoxide anion (O_2_^•−^), a type of ROS. In recent years, various studies had shown that members of the plant *rboh* gene family were involved in plant growth and developmental processes as well as in biotic and abiotic stress responses, but little is known about its functional role in upland cotton.

**Results:**

In the present study, 26 putative *Ghrboh* genes were identified and characterized. They were phylogenetically classified into six subfamilies and distributed at different densities across 18 of the 26 chromosomes or scaffolds. Their exon-intron structures, conserved domains, synteny and collinearity, gene family evolution, regulation mediated by *cis*-acting elements and microRNAs (miRNAs) were predicted and analyzed. Additionally, expression profiles of *Ghrboh* gene family were analyzed in different tissues/organs and at different developmental stages and under different abiotic stresses, using RNA-Seq data and real-time PCR. These profiling studies indicated that the *Ghrboh* genes exhibited temporal and spatial specificity with respect to expression, and might play important roles in cotton development and in stress tolerance through modulating NOX-dependent ROS induction and other signaling pathways.

**Conclusions:**

This comprehensive analysis of the characteristics of the *Ghrboh* gene family determined features such as sequence, synteny and collinearity, phylogenetic and evolutionary relationship, expression patterns, and *cis*-element- and miRNA-mediated regulation of gene expression. Our results will provide valuable information to help with further gene cloning, evolutionary analysis, and biological function analysis of cotton *rboh*s.

## Background

Plants are continually exposed to biotic and abiotic stresses, which negatively affect their growth and yield, causing enormous losses in agriculture worldwide. These stressors, such as pathogenic infections, drought, extreme temperatures and salt, lead to the over-accumulation of reactive oxygen species (ROS). ROS, including the superoxide anion (O_2_^·–^), hydroxyl radical (·OH), hydrogen peroxide (H_2_O_2_), singlet oxygen (^1^O_2_), ozone (O_3_) and nitric oxide (NO), have long been known to act as signal molecules in plants, regulating growth and development [1], programmed cell death (PCD) [2], hormone signaling [3], and responses to biotic and abiotic stresses [4, 5]. Excessive accumulation of ROS causes membrane damage, protein oxidation and DNA lesions, and can even lead to irreparable metabolic dysfunctions and cell death [6].

Plasma membrane NADPH oxidase (NOX) is a key enzyme involved in ROS formation. Plant NOX, known as respiratory burst oxidase homolog (rboh) and encoded by *rboh* genes, is a homolog of the mammalian NOX catalytic subunit known as gp91^phox^ [7]. The available crystal structures of classical plant rboh proteins have revealed the presence of two Ca^2+^-binding EF-hand motifs, six transmembrane domains and FAD- and NADPH-binding domains from the N-terminal region to the C-terminal region [8]. The plant *rboh* gene comprises a multiple gene family. In plants, *OsrbohA* was the first *rboh* gene identified in rice (*Oryza sativa* L.) [9], and subsequent studies indicated that different *rboh* genes in lower plants, monocots and dicots constituted a multigene family [10]. As more and more plant genomes are available, the *rboh* gene family has been characterized in some plant species, such as *Arabidopsis thaliana* (L.) Heynh [11], *O. sativa* L. [12], *Hordeum vulgare* L. [13], *Medicago truncatula* Gaertn. [14], *Vitis vinifera* L. [15], *Malus domestica* Mill. [16] and *Hevea brasiliensis* Muell. Arg. [17]. The genome of *A. thaliana* contains ten *Atrboh* genes, and it has been shown, by a meta-analysis of Genevestigator microarray datasets, that *AtrbohD* is the most highly expressed gene, whereas *AtrbohE* and *AtrbohH* show their highest expression in mature siliques, with very low expression in leaf tissues [12]. Expression of the *Atrboh* gene family is also induced in response to hormonal treatments and abiotic stresses. *AtrbohB* and *AtrbohE* show contrasting expression in response to the hormones abscisic acid (ABA), auxin and ethylene [12]. With the exception of heat stress conditions, under which all *Atrbohs* are found to be down-regulated, other abiotic stress conditions (drought, osmotic, salt, heat, cold, wounding, hypoxic and genotoxic) involve a mixture of up-and down-regulation of various *Atrbohs* [12, 18, 19]. In addition, the *Atrboh* gene family is also involved in regulating growth and development [1], and programmed cell death [2]. There are 9, 7 and 9 *rboh* genes in the genomes of rice, grape and apple, respectively, and the genome-wide analyses of *rboh* gene family in these plants reveal that the expression patterns of *rboh* genes varied under different treatments, indicating diverse functions in plant stress responses.

Allotetraploid upland cotton (*Gossypium hirsutum* L.) is both the world’s most important fiber crop as well as a source of seed oil and protein meal, and a model polyploid crop [20]. In a previous study, inhibiting the activity of the NADPH oxidase with diphenyleneiodonium (DPI) caused inhibition of both ROS formation and fiber cell elongation, a finding which reveals that NADPH oxidase is crucial for cotton fiber development [21]. However, a comprehensive characterization analysis of upland cotton *rboh* genes has not yet been reported, and no *rboh* gene of upland cotton has even been cloned. As cotton genomics develops, the release of the upland cotton genome sequence now allows a comprehensive genome-scale identification and analysis of *Ghrboh* genes [22–25]. In this study, we performed a genome-scale analysis of the *rboh* gene family in the upland cotton genome. Detailed information on genomic organization, gene structure, phylogenetic relationships and synteny with the diploid cotton *rboh* gene families were also reported. Furthermore, *cis*-elements in the putative promoters and microRNA (miRNA) target sites of *Ghrboh*s were analyzed, and the expression profiles of members of the *Ghrboh* gene family were investigated using RNA-Seq data and were analyzed using qPCR.

## Results

### Identification of *Rboh* genes in the upland cotton genome

To identify all the *rboh* genes in the upland cotton genome, HMMER and BLAST searches were performed using ten *rboh* genes from *A. thaliana* and conserved domains of rboh proteins as the queries. A total of 26 putative *Ghrboh* genes were identified. The distribution and density of *Ghrboh* genes on chromosomes (scaffolds) was not uniform. 18 chromosomes (scaffolds) carried *Ghrboh* genes, with 12 (chromosomes A1, A3, A8, A11, A12, D1, D8, D11, scaffold413_A2, scaffold3396_A12, scaffold3404_A12 and scaffold4588_D12) each carrying 1 *Ghrboh* gene and 4 (chromosomes A5, D3, D5 and D12) possessing 2 *Ghrboh* genes each, while the other 2 (chromosomes A7 and D7) involved each contained 3 *Ghrboh* genes. Additionally, half of the 26 *Ghrboh* genes were evenly distributed among Dt chromosomes (from tetraploid D) and At chromosomes (from tetraploid A). According to their localization in the *G. hirsutum* genome, we named these genes *Ghrboh1*–*26*, and the gene names, sequence IDs and genomic positions are shown in Table 1.

**Table 1:** The details of upland cotton *rboh* genes and proteins, containing physico-chemical and biochemical properties

Gene ID	Locus ID	Chromosome Position	Transcript Features			Protein Physicochemical Characteristics					
			ORF	ORF GC	Exon	Protein Length (aa)	Molecular Weight (kDa)	Isoelectric Point (*pI*)	GRAVY	Instability index (II)	Subcellular
			Length (bp)	Content (%)	Number						Prediction
*Ghrboh1*	Gh_A01G0943	A01:24768615–24,772,247 +	2574	44.9	12	857	97.03	8.84	−0.29	42.35	PM
*Ghrboh2*	Gh_D01G0990	D01:17844890–17,848,513 +	2823	44.1	10	940	107.08	8.97	−0.22	42.59	PM
*Ghrboh3*	Gh_A02G1791	scaffold413_A02:53689–60,746 -	2793	44.4	14	930	106.01	9.28	−0.22	48.63	PM
*Ghrboh4*	Gh_D03G0688	D03:24061389–24,068,512 +	2790	44.4	14	929	105.87	9.33	−0.21	48.87	PM
*Ghrboh5*	Gh_A03G0476	A03:10416355–10,426,420 +	2715	42.9	12	904	102.66	8.65	−0.28	39.13	PM
*Ghrboh6*	Gh_D03G1062	D03:35789027–35,795,976 -	2760	43.4	12	919	103.84	8.82	−0.26	38.91	PM
*Ghrboh7*	Gh_A05G1666	A05:17356320–17,359,403 +	2166	45.2	10	721	81.22	9.29	−0.19	42.68	PM
*Ghrboh8*	Gh_D05G1864	D05:17016832–17,023,628 +	2742	45.8	12	913	102.57	9.07	−0.27	42.08	PM
*Ghrboh9*	Gh_A05G2211	A05:25562865–25,570,955 +	2550	47.2	11	849	94.75	9.19	−0.30	36.86	PM
*Ghrboh10*	Gh_D05G2471	D05:24821383–24,825,199 +	2799	46.2	11	932	105.01	9.06	−0.30	38.39	PM
*Ghrboh11*	Gh_A07G0143	A07:1792736–1,798,327 -	2262	43.2	11	753	85.61	9.18	−0.12	50.56	PM
*Ghrboh12*	Gh_D07G0136	D07:1438083–1,443,321 -	2262	43.2	11	753	85.60	9.14	−0.10	49.82	PM
*Ghrboh13*	Gh_A07G0398	A07:5035106–5,042,836 +	2724	42.8	11	907	102.93	9.23	−0.28	39.25	PM
*Ghrboh14*	Gh_D07G0463	D07:4945697–4,952,499 +	2724	42.8	11	907	102.70	9.00	−0.24	40.70	PM
*Ghrboh15*	Gh_A07G0856	A07:14801554–14,805,328 -	2526	42.3	13	841	95.92	9.01	−0.31	43.98	PM
*Ghrboh16*	Gh_D07G0928	D07:12164885–12,168,659 -	2526	42.2	13	841	95.93	9.07	−0.32	42.36	PM
*Ghrboh17*	Gh_A08G0982	A08:68625165–68,666,345 -	2256	44.3	15	751	86.45	9.63	−0.14	44.52	PM
*Ghrboh18*	Gh_D08G1257	D08:41136123–41,143,012 -	2769	45.4	14	922	105.20	9.28	−0.22	46.29	PM
*Ghrboh19*	Gh_A11G2426	A11:82479840–82,484,514 +	2655	45.1	12	884	100.74	9.22	−0.31	42.52	PM
*Ghrboh20*	Gh_D11G2743	D11:56977773–56,982,441 +	2655	45.0	12	884	100.83	9.19	−0.316	43.32	PM
*Ghrboh21*	Gh_A12G1774	A12:80045991–80,051,555 -	2757	44.5	12	922	104.03	9.16	−0.31	41.92	PM
*Ghrboh22*	Gh_D12G1932	D12:52224716–52,230,284 -	2757	44.5	12	918	104.16	9.18	−0.30	40.90	PM
*Ghrboh23*	Gh_A12G2653	scaffold3396_A12:21385–29,027 +	2412	43.2	14	803	91.07	9.11	−0.09	45.39	PM
*Ghrboh24*	Gh_D12G1807	D12:50585049–50,592,640 -	2409	42.9	14	802	90.83	8.99	−0.08	43.79	PM
*Ghrboh25*	Gh_A12G2669	scaffold3404_A12:26809–33,492 -	2790	44.7	14	929	105.81	9.29	−0.23	45.69	PM
*Ghrboh26*	Gh_D12G2750	scaffold4588_D12:62625–69,256 +	2787	44.6	14	928	105.74	9.35	−0.24	45.66	PM

aa: amino acid; GRAVY: grand average of hydropathy, the GRAVY value for a peptide or protein is calculated as the sum of hydropathy values of all the amino acids, divided by the number of residues in the sequence, The greater the negative GRAVY, the better the hydrophilicity, and the greater the positive GRAVY, the stronger the hydrophobicity; The instability index (II) is a protein measurement that is used to determine whether the protein will be stable in a test tube (≤40, probably stable; > 40, probably not stable). PM: Plasma membrane.

### Sequence analysis and functional annotation

**Fig. 1:**
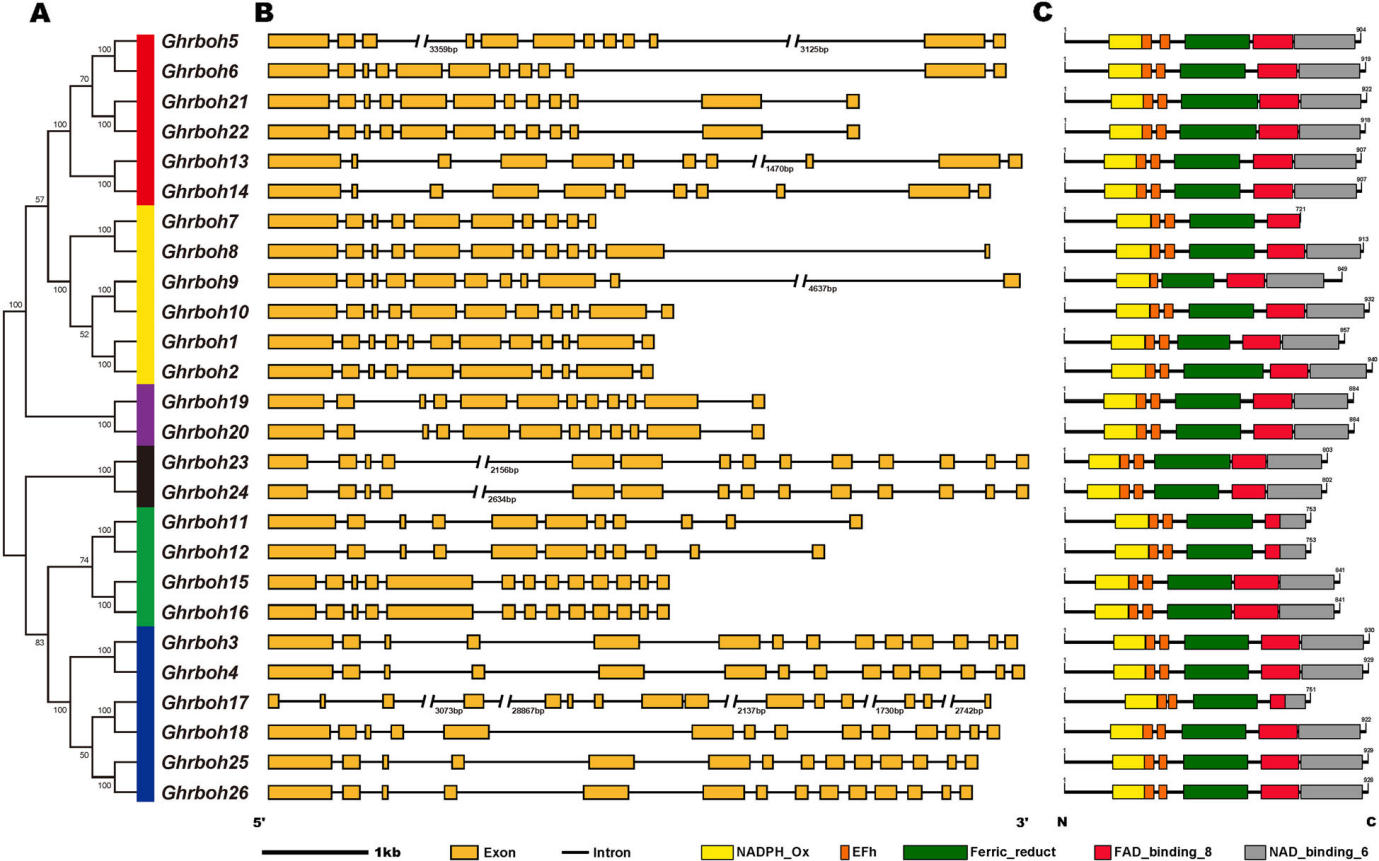
Cluster analysis, gene structure and domain analysis of upland cotton *rboh* gene family. (**A**) Phylogenetic tree of *G. hirsutum* rbohs constructed with MEGA 6.0 by the NJ method. Bootstrap values from 1000 replicates are indicated at each branch. Group I to VI represented by red, yellow, purple, black, green, and blue, respectively. (**B**) Exon–intron structures of *Ghrboh* genes. Yellow boxes and black horizontal lines indicated exons and introns, respectively. (**C**) Domain compositions of upland cotton rbohs. Only major domains were presented here based on our database searches in Pfam database (http://pfam.xfam.org/)

The result of *Ghrboh* gene structure analysis revealed that the numbers of exons in each gene varied between 10 and 15, with the lowest numbers of exons being in *Ghrboh2* and *Ghrboh7*, and the highest number in *Ghrboh17*. The genes clustering into the same group showed similar gene structures (Fig. 1a and b). Among the upland cotton *rboh* gene family, the order and approximate sizes of the exons were relatively conserved, compared with the more variable size of the introns (Fig. 1b). For instance, the spacing between the third and fourth exon of *Ghrboh17*, as well as between the fourth and fifth exon, was particularly variable, as seen in the corresponding exons of *Ghrboh5*, *Ghrboh9*, *Ghrboh13*, *Ghrboh23* and *Ghrboh24*. The results were consistent with those previously reported in *Arabidopsis*, barley, rice and grape [12, 13, 15].

The physico-chemical analysis of the predicted Ghrboh proteins encoded by candidate *Ghrboh* genes showed that the lengths, molecular masses, isoelectric points and instability indices of rboh proteins were within the ranges of 721–940 amino acids (aa), 81.22–107.08 kDa, 8.65–9.63 and 36.86–50.56, respectively (Table 1). All the predicted upland cotton rboh protein were alkaline. Other than Ghrboh5, Ghrboh6, Ghrboh9, Ghrboh10 and Ghrboh13, most predicted Ghrboh proteins were unstable (Table 1). Computational prediction of protein localization indicated that all Ghrboh proteins were localized in the plasma membrane. The information in the literature indicated that Ghrboh proteins were localized to the plasma membrane and transferred electrons from cytosolic NAD(P) H to an electron acceptor and catalyzed the formation of apoplastic O_2_^•−^ [9]. This corroborated our findings.

The conserved domains of candidate Ghrboh protein sequences were analyzed (Table 1). Although the Ghrboh proteins were of different sizes, their major functional domains were similar. Based on the domain analysis, all 26 predicted Ghrboh proteins contained one NADPH_Ox domain (PF08414), two elongation factor (EF)-hand motifs (PF00036), one Ferri_reduct domain (PF01794), one FAD-binding_8 domain (PF08022) and one NAD-binding_6 domain (PF08030) from N-terminus to C-terminus, except for Ghrboh9, which contained only one EF-hand motifs (Fig. 1c).

### Synteny and collinearity analysis

**Fig. 2:**
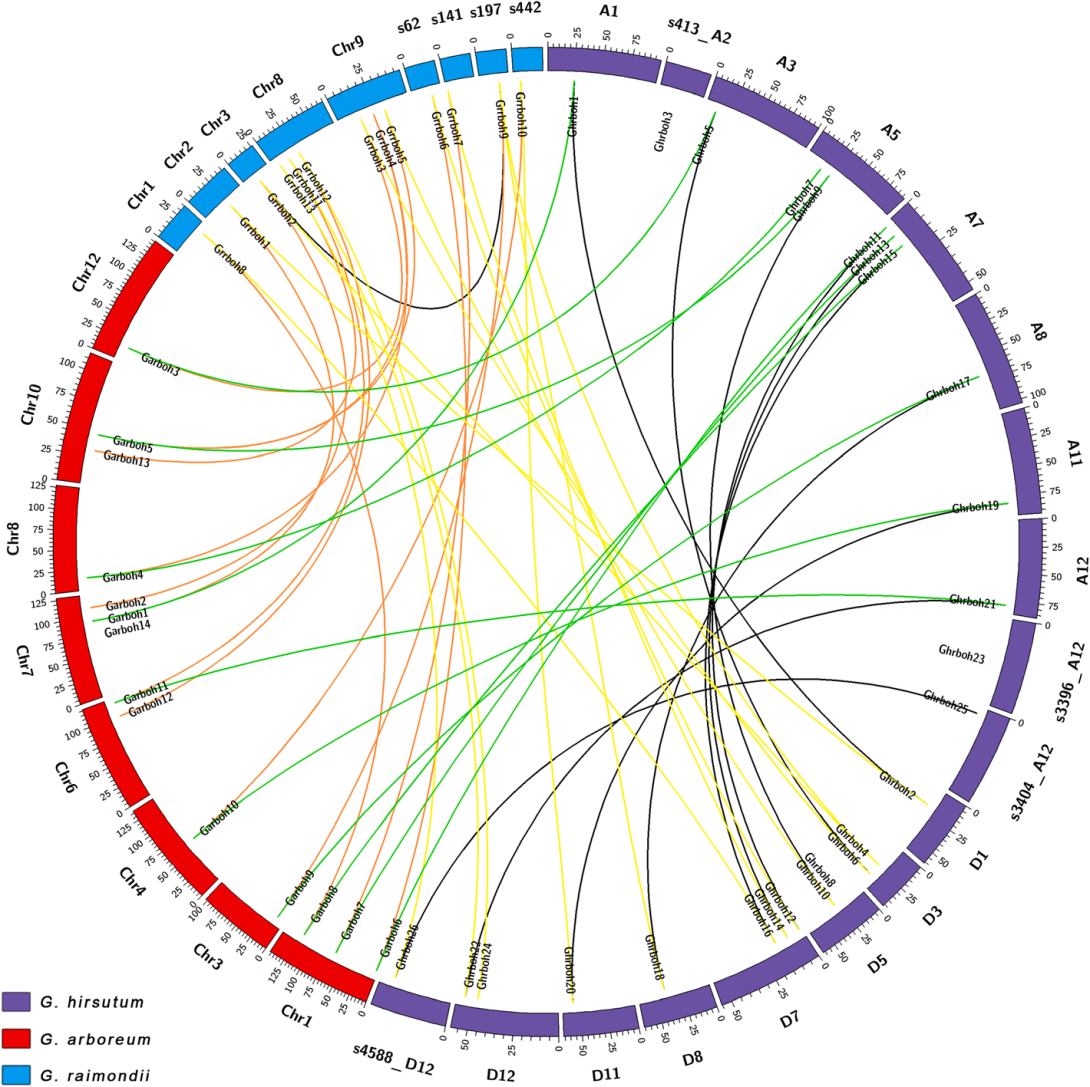
Chromosomal location and synteny relationships of *rboh* genes from *G. hirsutum*, *G. raimondii* and *G. arboretum*. *G. hirsutum*, *G. raimondii* and *G. arboretum* chromosomes are indicated in purple, blue and red, respectively. The putative orthologous *rboh* genes between *G. hirsutum* and *G. raimondii*, *G. hirsutum* and *G. arboretum*, and *G. raimondii* and *G. arboretum* are connected by yellow, red and orange lines, respectively. Black lines connect the putative paralogous genes. s413_A2, s, scaffold

To analyze the synteny and collinearity relationships of cotton *rboh* genes, we identified the orthologous and paralogous genes among *G. hirsutum*, *G. raimondii* and *G. arboreum* (Fig. 2, Additional file 1: Table S1 and Additional file 1: Table S2). From a *Gossypium* evolutionary point of view, we can deem that one *rboh* gene in the diploid species *G. raimondii* corresponds to 1 homologous gene in *G. arboreum* and 2 homologs, one each from the At and Dt subgenomes, in tetraploid *G. hirsutum*. We found that, of all 26 *rboh* genes identified in the *G. hirsutum* genome, 22 *Ghrboh*s had orthologs in *G. raimondii* and *G. arboreum*, with 10 showing an A genome origin and 12 D genome origin. Of 14 *Garboh* genes, 13 had orthologs in *G. raimondii* (Fig. 2 and Additional file 1: Table S1). The results indicated that the A- and D-subgenomes evolved independently after polyploid formation.

We further identified gene losses in syntenic blocks, among the cotton *rboh* genes that had no orthologs. *Ghrboh3*/*23* and *Ghrboh8* had no orthologs in *G. raimondii* and *G. arboreum*, respectively, *Garboh2*/*12*/*13*/*14* had no orthologs in *G. hirsutum*, *Grrboh4* had no orthologs in *G. hirsutum*, and *Garboh14* had no orthologs in *G. hirsutum* or *G. raimondii*. Considering the evolutionary history of cotton [24, 25], we hypothesized that the orthologous gene of *Garboh14* in *G. raimondii* was lost during divergence between *G. raimondii* and *G. arboreum* from their common ancestor (approximately 2~13 million years ago, MYA), and the orthologs of *Garboh2*/*12*/*13* in *G. hirsutum* were lost when the allotetraploid was formed approximately 1~1.5 MYA. These results indicated that more genes were lost from the At subgenome than from the Dt subgenome during the formation of *G. hirsutum*, which was consistent with the findings of a previous study [22]. Apart from gene loss, the result might also be artefacts, resulting from the sequencing methods used and genome assembly quality in different cotton species, or from errors of assembly and annotation in partial chromosomal regions. This possibility needs further investigation.

Gene duplications, occurring during the course of cotton evolution, have led to the development of new gene functions [26]. Genes might be duplicated by mechanisms other than whole-genome duplication (WGD), such as tandem, proximal and/or dispersed duplications, each of which might make different contributions to evolution [27]. To analyze the relationship between cotton *rboh* genes and gene duplication events, we characterized ten pairs of paralogous genes in the *G. hirsutum* genome, and one pair in the *G. raimondii* genome (Fig. 2 and Additional file 1: Table S2) and classified the duplicate genes. The duplicate genes of the *Ghrboh* gene family could be classified into WGD/segmental or dispersed duplicates. With the exception of *Ghrboh3*/*8*/*23*, which were dispersed duplicates, the rest of the *Ghrboh* genes were WGD/segmental duplicates, with tandem duplications not being observed. WGD/segmental duplicates were inferred by the presence of anchor genes in collinear blocks, whereas dispersed duplicates were paralogs that were neither near one another on chromosomes, nor did they show conserved synteny. These results indicated that WGD/segmental duplications mainly contributed to the expansion of the *Ghrboh* gene family in upland cotton.

### Phylogenetic and evolutionary analysis

**Fig. 3:**
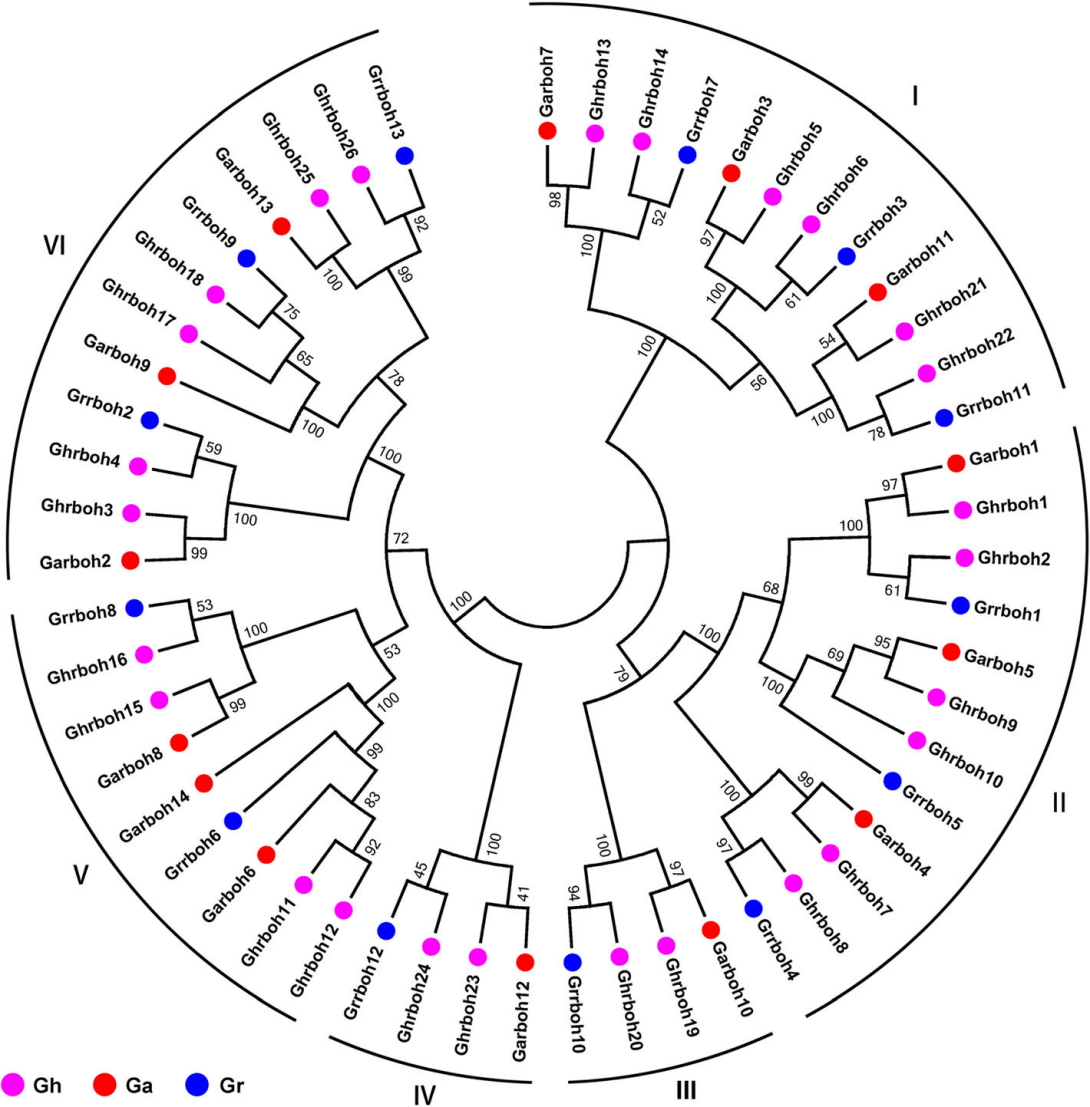
Neighbor-joining (NJ) phylogenetic tree of the *rboh* gene family among *Gossypium*. The tree was constructed with predicted full length rboh amino acid sequences from in *G. hirsutum* (Gh), *G. arboreum* (Ga), and *G. raimondii* (Gr)

To investigate the evolutionary relationships between rboh proteins among upland cotton and other *Gossypium* spp., 2 phylogenetic trees were independently constructed using predicted full-length amino acid sequences and the MEGA 6.0 software with the neighbor-joining (NJ) method (Fig. 3 and Fig. 1a). The *rboh* genes of 3 cotton species were clustered into 6 groups, which showed accordance with previous phylogenetic analyses of plant rbohs [15, 28]. Groups I to VI are represented by red, yellow, purple, black, green, and blue, respectively (Fig. 1a).

Using the same method as used to identify *rboh* genes in the upland cotton genome, we also searched for *rboh* genes in the genomes of lower aquatic to higher terrestrial plants. Among green alga, four *Crrboh*s were identified from *Chlamydomonas reinhardtii* P.A. Dangeard, but there were no rboh genes in the genome of the other green alga investigated, namely *Micromonas pusilla* (R. W. Butcher) I. Manton & M. Parke, *Ostreococcus lucimarinus* and *Volvox carteri* F.Stein. 4 *Pprboh*s were identified from the moss, *Physcomitrella patens* (Hedw.) Bruch & Schimp. In the spikemoss, *Selaginella moellendorffii* Hieron., a member of the Pteridophyta, there were 10 *Smrboh* genes. In the genome of the understory shrub *Amborella trichopoda* Baill., 6 *Amtrboh*s were identified. Among monocots, the number of *rboh*s was 7 in *Ananas comosus* (L.) Merr., 9 each in *Brachypodium distachyon* (L.) P.Beauv. and *O. sativa* L., and 10 each in *Sorghum bicolor* (L.) Moench and *Musa acuminata* Colla. Among eudicots, the number of *rboh*s was 7 in each of *Theobroma cacao* L., *Medicago truncatula* Gaertn. and *V. vinifera* L., 10 each in *A. thaliana*, *Malus domestica* Borkh. and *Daucus carota* L., 13 in *G. raimondii*, and 14 in *G. arboretum* (Additional file 1: Table S3). Evolutionary analysis using 20 species from lower aquatic to higher terrestrial plants showed that *rboh* genes first appeared in the green algae (*C. reinhardtii*) and the number of genes increased dramatically in pteridophytes (*S. moellendorffii*), then stayed relatively stable until the upland cotton evolved (Additional file 1: Figure S1). This finding was consistent with a WGD event resulting in tetraploid cotton after two diploid cotton species reunited geographically around 1~2 MYA [29].

In terms of *Gossypium rboh*s, the total number in *G. raimondii* and *G. arboretum*, which were considered to be the A-genome ancestor and D-genome ancestor, respectively, of *G. hirsutum*, was 27, which was nearly equal to that in *G. hirsutum*. All other upland cotton *rboh* genes were clustered together as either *G. raimondii* or *G. arboretum rboh* genes. This finding was consistent with the hypothetical origins and history of allotetraploid cotton [29].

In addition, to calculate the evolutionary time of *Ghrboh* genes and gain more insights into the divergence of the upland cotton *rboh* gene family after polyploidization, an estimation of their non-synonymous (*Ka*) and synonymous (*Ks*) nucleotide substitutions and their ratio (*Ka*/*Ks*) during evolution were calculated using the add_ka_and_ks_to_collinearity.pl program of MCScanX software (Additional file 1: Table S2). The *Ka*/*Ks* ratio is a measure used to examine the mechanisms of gene duplication evolution after divergence from an ancestor and to estimate the balance between neutral selection (*Ka*/*Ks* = 1), purifying selection (*Ka*/*Ks* < 1) and positive selection (*Ka*/*Ks* > 1) [30]. The analysis demonstrated that nine of the ten *Ghrboh* paralogous pairs had *Ka*/*Ks* ratios less than 1, indicating that the *Ghrboh* gene family had been influenced principally by high purifying selection, while one pair of duplicated genes had a *Ka*/*Ks* ratio greater than 1, implying that they had evolved under positive selection (Additional file 1: Table S2). This result revealed that the *Ghrboh* genes were evolving slowly and had conserved characteristics at the protein level. According to the neutral substitution (*r*) rate of 2.6 × 10^− 9^ synonymous mutations per locus per year, the estimated divergence time (*t*) was calculated from the equation “*t* = *Ks*/2*r*” MYA [31]. The ten paralogous pairs were calculated to have diverged between 3.32 MYA (*Ks* = 0.0173) and 16.88 MYA (*Ks* = 0.0878), with an average of 8.34 MYA (Additional file 1: Table S2). These results suggested that the expansion of *Ghrboh* genes in upland cotton mostly arose as a result of WGD/segmental events during the divergence of one common ancestor into *G. raimondii* and *G. arboreum* approximately 2~13 MYA [22].

### Expression profiles of *Ghrboh* genes in different tissues/organs and development stages

**Fig. 4:**
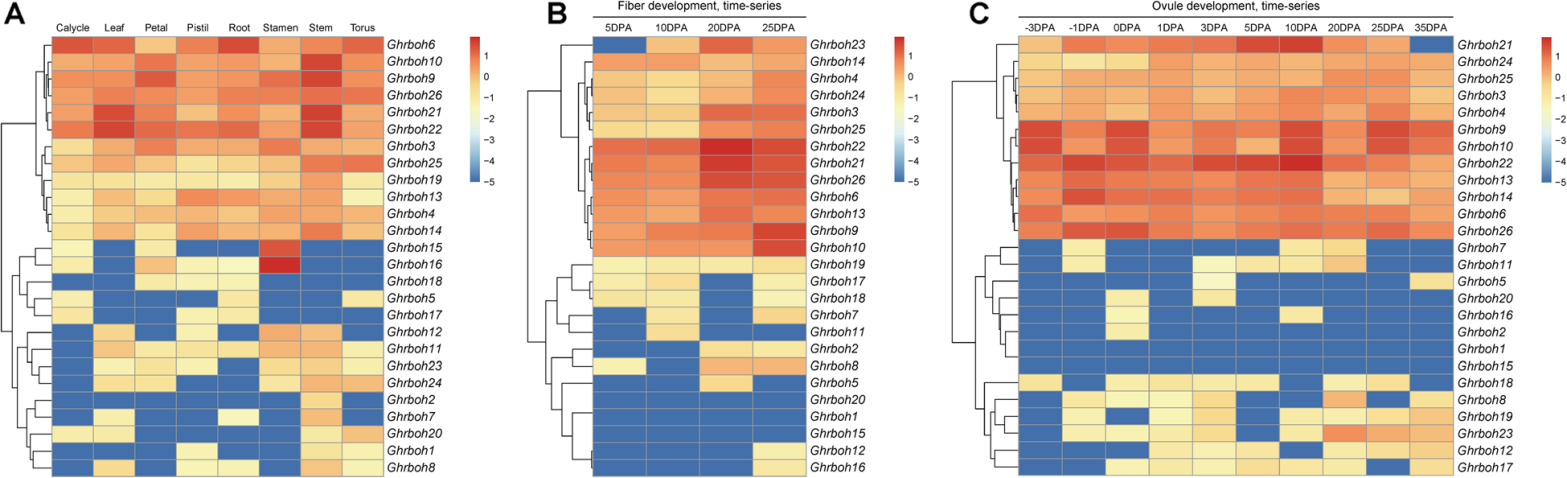
Expression profiles of *Ghrbohs* in different tissues/organs and development stages. The log_2_ of FPKMs values calculated by RNA-Seq data were shown as a heat map. The colors of the bar shown to the right of the heat-map varied from red to blue representing the relative expression levels from high to low. FPKMs data was obtained from ccNET (http://structuralbiology.cau.edu.cn/gossypium/) and CottonFGD (https://cottonfgd.org/). (**A**) The heat-map showed the hierarchical clustering of the relative expression of 26 *Ghrbohs* in root, stem, leaf, petal, torus, stamen, pistil, calycle. (**B**) The heat-map showed the hierarchical clustering of the relative expression of 26 *Ghrbohs* in fibers at 5, 10, 20 and 25 dpa. (**C**) The heat-map showed the hierarchical clustering of the relative expression of 26 *Ghrbohs* in ovules at − 3, − 1, 0, 1, 3, 5, 10, 20, 25 and 35 dpa

Gene expression profiles are closely associated with gene functions. Plant *rboh* genes are involved in growth and development [1], programmed cell death [2] and so on. To preliminarily study their biological functions in upland cotton with respect to different developmental processes, we initially collected the transcript profiles from root, stem, leaf, petal, torus, stamen, pistil, calycle, and ovules at − 1/0/1/3/5/10/20/25 days post anthesis (dpa) and fibers at 5/10/20/25 dpa from RNA-Seq data published by Zhang et al. using the *G. hirsutum* cultivar TM-1 [24] (Fig. 4).

Generally, the candidate *Ghrboh* genes showed very dynamic expression profiles in the afore-mentioned eight tissues and/or organs. Of the 26 candidate genes, six *Ghrboh* genes (*Ghrboh6*/*9*/*10*/*21*/*22*/*26*) were highly expressed in most of the eight tissues and/or organs, whereas the expression of a further 6 *Ghrboh* genes (*Ghrboh3*/*4*/*13*/*14*/*19*/*25*) were higher in some tissues and/or organs, but much lower or even barely detectable in others (Fig. 4a). For instance, the expression of *Ghrboh25* was higher in stem and torus, lower in leaf, and almost undetectable in the root, petal, stamen, pistil, and calycle. Furthermore, 12 *Ghrboh* genes (*Ghrboh1*/*2*/*5*/*7*/*8*/*11*/*12*/*17*/*18*/*20*/*23*/*24*) were expressed at very low levels or were even barely detectable in all eight tissues and/or organs tested. Remarkably, *Ghrboh15* and *Ghrboh16* were expressed constitutively in the stamen, but their expression levels in other tissues and/or organs were very low (Fig. 4a).

In addition to the tissue- or organ-specific expression profiles of *Ghrboh*s, we also analyzed the expression of all candidate *Ghrboh* genes during the cotton fiber and ovule development processes, using microarray expression data (Fig. 4b and Fig. 4c). The results showed that not all the candidate *Ghrboh* genes were expressed at the different developmental stages of upland cotton fibers and ovules. Overall, there were two types of fiber/ovule gene expression profiles: those which were expressed more-or-less constitutively, and those which were expressed at extremely low or even undetectable levels during the process of fiber and ovule development. Another interesting scenario was that, during fiber development, *Ghrboh1*/*15*/*20* did not show any detectable expression, whereas three *Ghrboh* genes (*Ghrboh21*/*22*/*26*) showed expression levels at 20 dpa between 2- and 6-fold higher than those observed at other stages, while *Ghrboh9*/*10* exhibited continuous increases in expression throughout fiber development (Fig. 4b). During ovule development, *Ghrboh1*/*15* did not show any detectable expression, *Ghrboh21* and *Ghrboh22* were down-regulated at the stage from 10 dpa to 20 dpa, *Ghrboh9* was down-regulated at the stage from 0 dpa to 1 dpa and from 10 dpa to 20 dpa, and *Ghrboh10* was up-regulated at the stage from 5 dpa to 10 dpa and down-regulated at the stage from 10 dpa to 20 dpa (Fig. 4c).

Since NADPH oxidase is crucial for cotton fiber development [21], gene expression patterns in fibers and ovules at different time points after flowering were studied using real-time quantitative (qPCR) (Additional file 1: Figure S2 and Additional file 1: Figure S3). We used the transcript levels of the *Ghrboh* genes in the young leaves (YL) as references and set the reference value to 1. The qPCR analysis revealed that all of the 26 *Ghrboh* genes were differentially expressed in each of the developmental stages of fiber (5, 10, 20 and 25 dpa) and ovule (− 1, 0, 3, 5, 10, 20 and 25 dpa) tested in upland cotton. During fiber development, 4 *Ghrboh* genes (*Ghrboh10*/*14*/*25*/*26*) were significantly differentially expressed at 5 dpa, 3 *Ghrboh* genes (*Ghrboh3*/*18*/*20*) were significantly differentially expressed at 10 dpa, 3 *Ghrboh* genes (*Ghrboh4*/*9*/*17*) were significantly differentially expressed at 5 and 10 dpa, 3 *Ghrboh* genes (*Ghrboh8*/*12*/*15*) were significantly differentially expressed at 10 and 20 dpa, *Ghrboh13* and *Ghrboh22* were significantly differentially expressed at 5 and 20 dpa, *Ghrboh24* was significantly differentially expressed at 5 and 25 dpa, and *Ghrboh21* was significantly differentially expressed at 5, 10 and 20 dpa (Additional file 1: Figure S2).

During ovule development, 6 *Ghrboh* genes (*Ghrboh2*/*3*/*4*/*8*/*17*/*19*) were significantly differentially expressed at − 1 and 0 dpa, 5 *Ghrboh* genes (*Ghrboh7*/*11*/*15*/*16*/*18*) were significantly differentially expressed at 0 dpa, 3 *Ghrboh* genes (*Ghrboh13*/*14*/*21*) were significantly differentially expressed at 0, 5 and 10 dpa, *Ghrboh12* was significantly differentially expressed at − 1 and 5 dpa, *Ghrboh23* was significantly differentially expressed at 0 and 25 dpa, *Ghrboh22* was significantly differentially expressed at 3 and 5 dpa, and *Ghrboh24* was significantly differentially expressed at 5 and 10 dpa (Additional file 1: Figure S3). These qPCR results will lay the groundwork for further cloning and functional analysis of the *Ghrboh* gene family.

All these results indicated that expression of the members of the *Ghrboh* gene family exhibited temporal and spatial specificity and might be involved in the growth and development of different tissues or organs of upland cotton.

### Expression profiles of *Ghrboh* genes under different abiotic stress treatments

**Fig. 5:**
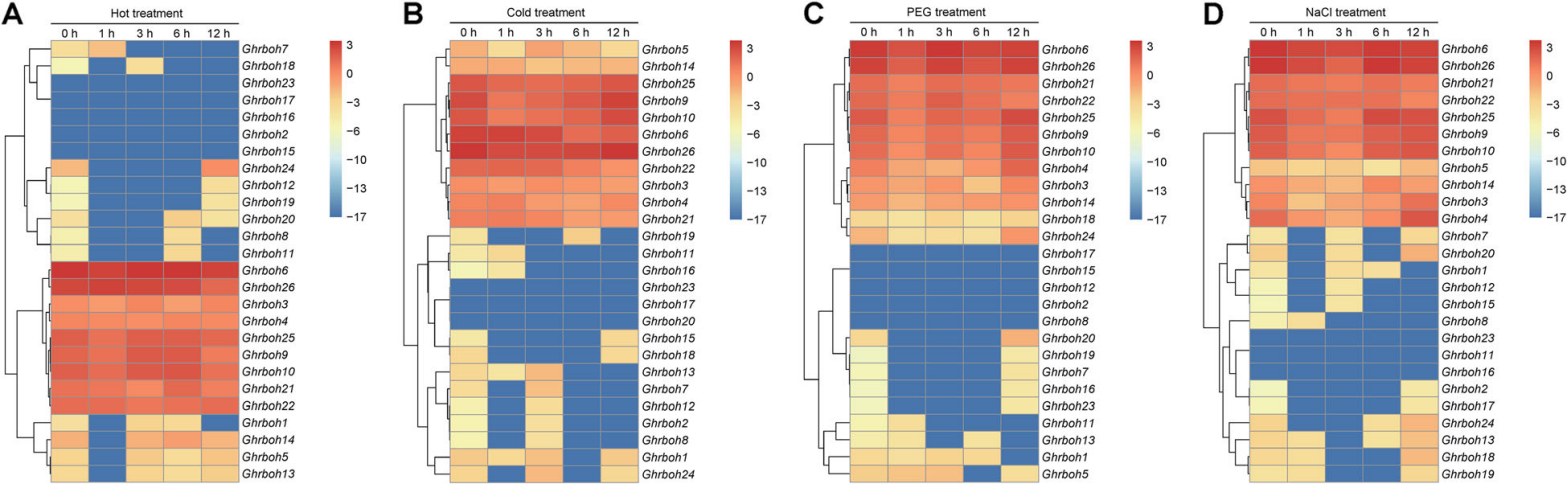
Expression profiles of *Ghrbohs* under different stress treatments. The log_2_ of FPKMs values calculated by RNA-Seq data were shown as a heat map. The colors of the bar shown to the right of the heat-map varied from red to blue representing the relative expression levels from high to low. FPKMs data was obtained from ccNET (http://structuralbiology.cau.edu.cn/gossypium/) and CottonFGD (https://cottonfgd.org/). The heat-map showed the hierarchical clustering of the relative expression of *Ghrbohs* under hot, cold, PEG and salt treatments (**A**-**D**)

Previous studies had revealed that plant *rboh* genes were widely associated with abiotic stress responses under normal and stressed growth conditions [4, 11]. To determine whether the *Ghrboh* genes responded to stress conditions, we examined the expression profiles of all 26 predicted *Ghrboh* genes in response to a series of abiotic stresses (hot, cold, drought (polyethylene glycol, PEG) and salt), using RNA-Seq data (Fig. 5) and qPCR (Additional file 1: Figure S4). As shown in Fig. 5, the upland cotton *rboh* gene family was differentially expressed in the leaves under hot, cold, drought and salt stress conditions. Under high-temperature stress treatment, a total of 9 genes (*Ghrboh3*/*4*/*6*/*9*/*10*/*21*/*22*/*25*/*26*) showed continuous and stable expression, whereas *Ghrboh2*/*15*/*16*/*17*/*23* did not show any detectable expression, suggesting that they were not involved in heat-stress response. In addition, *Ghrboh1*/*5*/*13*/*14* were down-regulated at the 1 h time point of the heat stress treatment (Fig. 5a). Under cold stress treatment, a total of 11 genes (*Ghrboh3*/*4*/*5*/*6*/*9*/*10*/*14*/*21*/*22*/*25*/*26*) showed continuous and stable expression, whereas expression of *Ghrboh17*/*20*/*23* was not induced by cold treatment, and a total of 7 genes were down-regulated at early time points and up-regulated after experiencing a longer cold treatment period (Fig. 5b). Under PEG treatment, a total of 12 genes were expressed continuously and stably, among which *Ghrboh6*/*9*/*10*/*21*/*22*/*25*/*26* showed higher expression, and *Ghrboh3*/*4*/*14*/*18*/*24* showed lower expression. *Ghrboh2*/*8*/*12*/*15*/*17* were not induced by PEG treatment, and a total of 5 genes were down-regulated at early treatment time points and up-regulated after experiencing a longer PEG treatment (Fig. 5c). Under salt treatment, a total of eleven genes were expressed continuously and stably, among which *Ghrboh6*/*9*/*10*/*21*/*22*/*25*/*26* exhibited higher expression, and *Ghrboh3*/*4*/*5*/*14* showed lower expression. *Ghrboh11*/*16*/*23* were not induced by salt treatment, and *Ghrboh1*/*2*/*7*/*8*/*12*/*15*/*17*/*20* were expressed at extremely low or even undetectable levels during salt treatment (Fig. 5d).

To determine gene expression under abiotic stress, expression in leaf tissue from plants exposed to salinity or drought conditions was determined using qPCR. The results showed that the expression patterns of *Ghrboh*s were complex under salinity or drought treatments. Under salinity stress, 4 *Ghrboh* genes (*Ghrboh9*/*14*/*21*/*22*) were up-regulated and maintained a relatively high expression level, with *Ghrboh14* and *Ghrboh22* exhibited approximately 3- to 30-fold induction. In contrast, expression of 3 genes (*Ghrboh4*/*15*/*24*) was down-regulated in response to salinity stress. 3 genes (*Ghrboh3*/*10*/*23*) were up-regulated at 6 h, 3 genes (*Ghrboh10*/*13*/*20*) were up-regulated at 12 h, 5 genes (*Ghrboh2*/*8*/*11*/*16*/*17*) were up-regulated at 6 h and 12 h. *Ghrboh18* and *Ghrboh19* were significantly down-regulated at 3 h, and significantly up-regulated over the rest of the NaCl treatment. The results suggested that these genes, acting as positive or negative regulators, were involved in the response of upland cotton to salinity stress (Additional file 1: Figure S4).

Under drought stress, 12 genes (*Ghrboh2*/*5*/*6*/*8*/*9*/*10*/*11*/*16*/*19*/*21*/*22*/*23*) were down-regulated in response to drought stress. In contrast, *Ghrboh3* and *Ghrboh7* were up-regulated and exhibited approximately 2- to 20-fold induction. In addition, 5 genes (*Ghrboh1*/*14*/*17*/*18*/*24*) were significantly up-regulated at 3 h, 2 genes (*Ghrboh25*/*26*) were significantly up-regulated at 1 h and 3 h, *Ghrboh4* was significantly up-regulated at 6 h, *Ghrboh15* was significantly up-regulated at 1 h and 12 h and significantly down-regulated or exhibited no significant expression differences over the rest of the PEG treatment (Additional file 1: Figure S4).

The reasons underlying the comprehensive expression profiles of these genes might indicate their vital functions in response to heat, cold, drought or salt treatment. The results of the expression analysis suggested that the *rboh* gene family of upland cotton may be important in terms of stress responses as well as developmental processes.

### *Cis*-element analysis of putative *Ghrboh* promoters

**Fig. 6:**
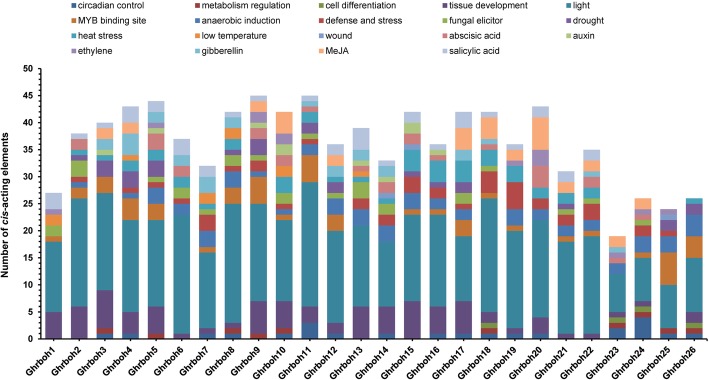
*Cis*-elements analysis of putative *Ghrboh* promoters. Different *cis*-elements with the same or similar functions were shown in the same color

We regarded the 1.5-kb genomic sequences upstream from the transcription start site (TSS) of each upland cotton *rboh* gene as putative promoter regions and used the PlantCARE tool to identify the presence of *cis*-elements which could be controlling the expression of the *Ghrboh* genes. All 26 putative *Ghrboh* promoters possessed the typical core *cis*-acting elements in promoter regions, including TATA and CAAT boxes. Potential regulatory *cis*-acting elements identified from the upstream region of the *Ghrboh* genes are shown in Fig. 6 and Additional file 1: Table S4. In addition to the TATA and CAAT boxes, there were 19 types of *cis*-acting elements, which could be grouped into four different functional categories, namely stress response, hormone regulation, cellular development, and metabolism regulation. These findings were consistent with those from a previous study in *Arabidopsis* and rice [12].

The results revealed that 7 types of stress-response elements, namely ARE, MBS, Box-W1, HSE, LTR, WUN-motif and TC-rich repeats, with responses to anaerobiosis, drought, fungal elicitors, heat stress, cold stress, wound stress, and defense stress, respectively, were identified in the *Ghrboh* promoter regions. Furthermore, 11 types of hormone regulation elements, namely ABRE, AuxRR-core, TGA-box, TGA-element, ERE, GARE-motif, TATC-box, P-box, CGTAC-motif, TGACG-motif and TCA-element, which were associated with abscisic acid (ABA), auxin (IAA), ethylene, gibberellin (GA), methyl jasmonate (MeJA) and salicylic acid (SA) responses, were found in the *Ghrboh* promoters.

In the cellular development category, 7 types of *cis*-elements, namely HD-Zip 1, HD-Zip 2, Skn-1_motif, CAT-box, RY-element, as-2-box and as1, which are associated with cell differentiation and tissue development, were identified in the *Ghrboh* promoter regions. In the metabolism regulation category, there were 4 types of elements, namely O_2_-site, MBSII, Unnamed_1 and circadian, which are associated with zein metabolism regulation, flavonoid biosynthesis gene regulation, phytochrome regulation and circadian control, respectively. In addition, many light-responsive elements were present in in each *Ghrboh* promoter. There were 29 different types of light-responsive elements and every putative promoter contained between six and 13 types (Fig. 6 and Additional file 1: Table S4). The putative promoters of *Ghrboh* genes carried different types and numbers of *cis*-regulatory elements, indicating that *Ghrboh* genes might be involved in some growth and development progresses, such as cotton fiber development, and were controlled by different regulatory mechanisms in response to various stresses.

### Predicting miRNA target sites

**Fig. 7:**
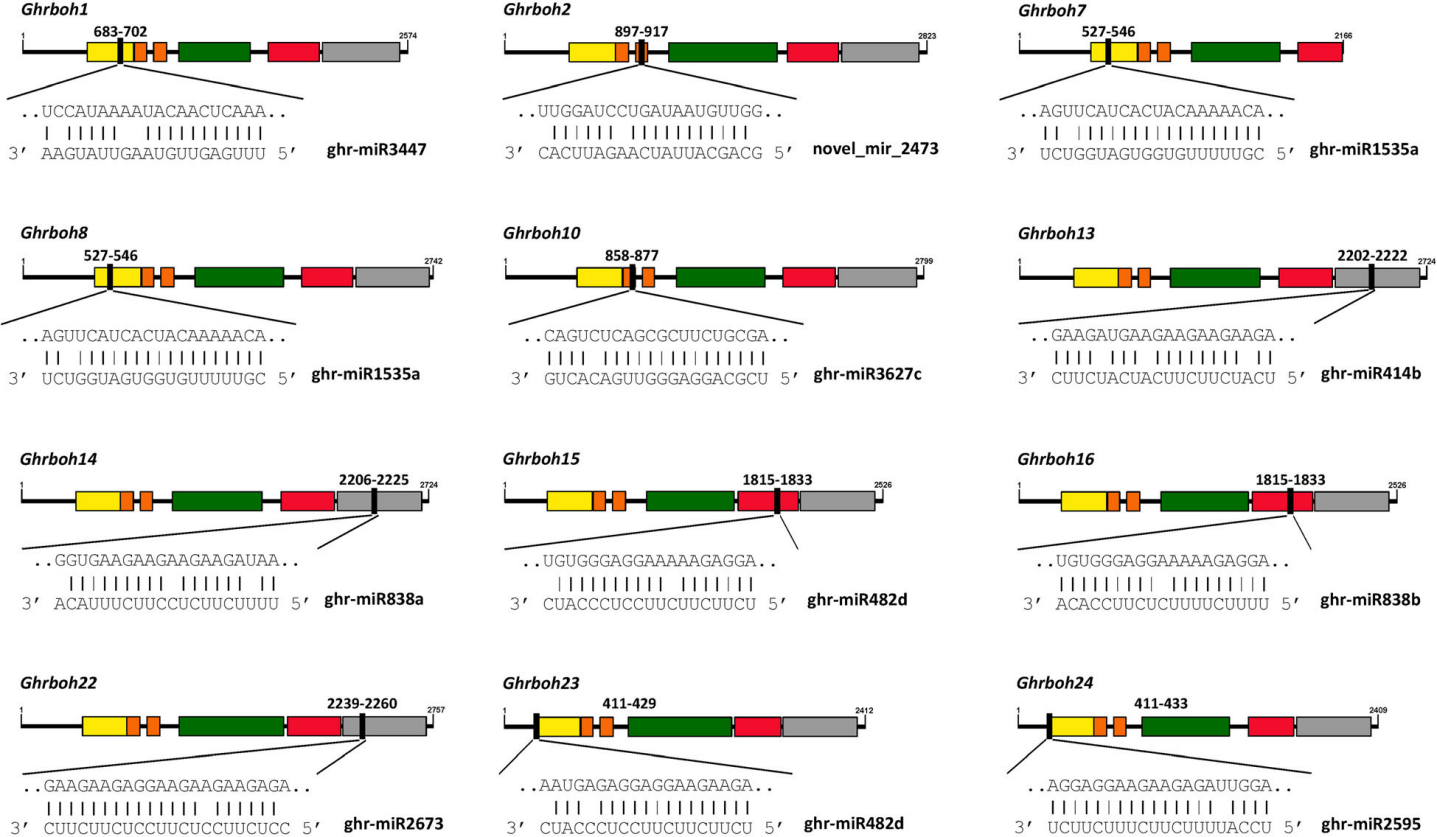
The prediction of targeting regulatory relations between *Ghrbohs* and *G. hirsutum* miRNAs. Black lines represented ORFs of *Ghrbohs*. NADPH_Ox domain, EF-hand motif, Ferri_reduct domain, FAD-binding_8 domain and NAD-binding_6 domain, were represented by boxes filled with yellow, orange, green, red and grey, respectively. miRNA complementary sites (black filling) with the nucleotide positions of *Ghrbohs* cDNAs were pointed out. The RNA sequence of each complementary site from 5′ to 3′ and the predicted miRNA sequence from 3′ to 5′ are shown in the expanded regions

To predict microRNA (miRNA)-mediated post-transcriptional regulation of *Ghrboh*s, we searched *Ghrboh*s coding sequences for target sites of *G. hirsutum* miRNAs, using the psRNATarget server with stricter parameters than default. The results showed that 15 *G. hirsutum* miRNAs targeted 17 *Ghrboh*s (Fig. 7, Additional file 1: Table S5). These miRNAs included conserved upland cotton miRNAs [32] and novel miRNAs identified by small-RNA sequencing and bioinformatics analysis [33, 34]. The results showed that *Ghrboh1* and *Ghrboh2* were both targeted by ghr-miR3447 and novel_miR_2473 with sites in the NADPH_Ox domain and the second EF-hand motif of the N-terminus, respectively; *Ghrboh7* and *Ghrboh8* were both targeted by ghr-miR1535a with sites in the NADPH_Ox domain; *Ghrboh10* was targeted by ghr-miR3627c with a site in the NADPH_Ox domain; ghr-miR414b and ghr-miR838a targeted *Ghrboh13* and/or *Ghrboh14* with a site in the NAD-binding_6 domain; ghr-miR482d and ghr-miR838b both targeted *Ghrboh15* and *Ghrboh16* with the same sites in the FAD-binding_8 domain; ghr-miR2673 targeted *Ghrboh21* and *Ghrboh22* with a site in the NAD-binding_6 domain; ghr-miR482d and ghr-miR2595 targeted *Ghrboh23* and *Ghrboh24* with a site in the NADPH_Ox domain (Fig. 7). In addition to the target sites described above, other, novel miRNAs of upland cotton targeted *Ghrboh*s. Mar-F-3-m0087 targeted *Ghrboh11* and *Ghrboh12* with a site in the NADPH_Ox domain; and Mar-F-2-m0069 and ghr-miR2949a targeted *Ghrboh17* and/or *Ghrboh18* with a site in the Ferri_reduct domain or FAD-binding_8 domain, respectively (Additional file 1: Table S5). Our prediction results revealed that the miRNA-mediated post-transcriptional regulation of *rboh*s might be conserved in *G. hirsutum*, and many researchers have studied *rboh* genes involved in the process of morphogenesis and development, and response to biotic and abiotic stress in plants [10], but there have been few reports of gene expression and regulation being mediated by miRNAs. These miRNAs, predicted to target *Ghrboh*s, resulted from computational predictions and deep sequencing, and they were reported to be involved in some biological processes reported in plants, including responses to environmental stresses and regulation of cell growth and development [32, 34–38]. The expression patterns of the miRNAs mentioned above and their targets need to be detected and verified in further experiments to confirm and determine their biological functions in upland cotton, and this is a topic on which we plan to report in greater detail in the future.

## Discussion

The plant *rboh* gene family has been comprehensively analyzed in *Arabidopsis*, rice, grape, apple and rubber tree, respectively. However, there had been no genome-scale analysis of the *rboh* gene family in upland cotton before the present study. In this study, the upland cotton *rboh* gene family was identified at the genome scale, and the expression patterns of individual members were analyzed.

### The *Rboh* gene family was expanding in upland cotton genome

We identified 26 putative *rboh* genes (*Ghrboh1* through *Ghrboh26*) from the genome of upland cotton cultivar TM-1. We also searched another 20 plant genomes for *rboh* genes, from lower aquatic to higher terrestrial plants, which were at key evolutionary nodes. The number of *rboh* genes in upland cotton is much larger than those from other plants, and the results showed that *rboh* gene family first appeared in green algae (*C. reinhardtii*, about four members) and the number dramatically increased in pteridophytes (*S. moellendorffii*, about ten members), then stayed relatively stable until the upland cotton evolved (*G. hirsutum*, having approximately twice the gene number of *S. moellendorffii* etc., and about six times the number of *C. reinhardtii*) (Additional file 1: Figure S1). The results showed that, as a result of plant evolution, the *rboh* gene family expanded.

Gene duplications, occurring during cotton evolution, have played a significant role in the expansion of the *rboh* gene family in the genome [26]. Genes may be duplicated by some mechanisms, such as WGD or polyploidy, tandem, proximal and/or dispersed duplication [27]. It is generally known that WGD or polyploidy are important processes throughout the history of plant evolution, and have long been recognized as fundamental mechanisms of diversification and gene family expansion in plants [39–42]. Throughout plant history, there have been some common WGD or polyploidy events, such as occurred at the appearance of the seed plants approximately 310 MYA and another paleohexaploidization event at the evolution of the eudicots 130~190 MYA, as well as some lineage-specific WGD or polyploidy events, such as the WGD series of the ρ-σ-τ in the cereal grass lineage and the α-β-γ series in the *Arabidopsis* lineage [43]. In the hypothetical origins and evolutionary history of dicotyledonous allotetraploid cotton, allopolyploid cotton may have appeared in the last 1~2 MYA, as a consequence of trans-oceanic dispersal of an A-genome taxon *G. arboreum* (A2) to the New World approximately 5~10 MYA, followed by hybridization with an indigenous D-genome diploid *G. raimondii* (D5), followed by chromosome doubling [29]. The results of the phylogenetic and evolution analysis showed that the paralogous pairs of *Ghrboh*s diverged approximately 8.34 MYA (Additional file 1: Table S2). The results of synteny and collinearity analysis revealed that the duplicate genes of the *Ghrboh* gene family were mainly duplicated by WGD or segmental duplications. These results suggested that the expansion of *Ghrboh* genes in upland cotton mostly arose from WGD or polyploidy events as one common ancestor diverged into *G. raimondii* and *G. arboreum* at approximately 2~13 MYA. Thus, we hypothesized that the common and lineage-specific WGD or polyploidy events and segmental duplications, which generated duplicate copies of plant *rboh* genes and were widespread throughout plant history, are the major factor responsible for the expansion of the *Ghrboh* gene family.

### *Ghrbohs* probably participate in cotton fiber development and stress response by mediating ROS production

There are various reports that suggest that NADPH oxidases mediate a multiplicity of physiological functions involved in development [2, 44, 45], adaptation to environment [46–48], and interactions with other organisms [49, 50], and the expression patterns of *rboh* genes have been determined in many plant species. In apple, *MdrbohD1*–*3* and *F* were expressed in leaves, in vitro shoot and suspension cell cultures, and expression of *MdrbohE2* and *H1*–*2* varied among the tissues. The *MdrbohD1*–*2* and *F* genes were involved in regulation of developmental processes of apple shoots and in response to oxidative stress damage [16]. In rice, under drought stress, the expressions of *OsNox1*–*3*, *OsNox5* and *OsNox9* were up-regulated, but the expression of *OsNox6* was down-regulated. Under high-temperature conditions, the expressions of *OsNox5*–*9* were up-regulated, but the expressions of *OsNox1*–*3* were significantly down-regulated. Under salt stress, the expressions of *OsNox2* and *OsNox8* were increased but the expressions of *OsNox1*/*3*/*5*/*6* decreased [28]. In grape, the expression levels of *VvrbohA*/*B*/*C1* were markedly induced by drought and salinity stresses. After powdery mildew inoculation, the expression of *VvrbohB*/*C2*/*D* increased while that of *VvrbohH* decreased [15]. These results suggest that the expression of plant *rboh* genes varied greatly with tissues and environmental conditions, suggesting diverse functions of *rboh* genes in the plant development and stress responses.

Although, cotton, which is a widely cultivated polyploid fiber crop, is a relatively salt and drought tolerant crop, exposure of cotton to high salinity or drought conditions can directly lead to a considerable negative impact on cotton growth and development and lint yield. To investigate the expression patterns of members of the *Ghrboh* gene family, we analyzed the transcript levels of all 26 *Ghrboh* genes in different organs/tissues, at different developmental stages, and following exposure to some abiotic stresses. From the results of transcriptomic data and qPCR, we found that the expression patterns of the *rboh* gene family of upland cotton exhibited diverse and complex stress-response expression signatures, which may be important both for stress responses and developmental processes.

To preliminarily explore which member(s) contributed mainly to the stress response or developmental processes, we analyzed the differential expression of the *Ghrboh* gene family in different tissues/organs, at different developmental stages of fiber/ovule, and under different abiotic stresses. Based on RNA-Seq data, the statistical significance of difference of gene expression was assessed with log2 (fold-change of *Ghrbohs* FPKMs (fragments per kilobase of transcript per million mapped reads)) ≥1 and a *p*-value < 0.05 (Fisher’s Exact Test). It is worth noting that 3 genes (*Ghrboh10*/*21*/*22*) were significantly up-regulated during fiber and ovule development, 1 gene (*Ghrboh26*) was specifically responsive to salt stress and significantly expressed during fiber development, 1 gene (*Ghrboh9*) was significantly up-regulated during ovule development, and 6 genes (*Ghrboh6*/*13*/*14*/*15*/*16*/*25*) were significantly expressed in different tissues and organs (Additional file 1: Table S6 and Additional file 1: Figure S5).

The results of qPCR showed that some *Ghrboh* genes were specifically expressed at certain time points of fiber/ovule development and significantly induced by salt/drought stresses (Additional file 1: Figure S2 and Additional file 1: Figure S3). The results of digital expression were basically consistent with the results of the qPCR studies, although there were differences in the expression levels between our qPCR results and the RNA-Seq data. The differences might be because the material was collected from different tissues of different varieties at different growth stages. The material for qPCR was taken from *G. hirsutum* L. cv. SF06 at the appropriate stage, while the material for the RNA-seq was the leaf tissue of TM-1 (the age is not applicable). Despite this discrepancy, the findings suggested that these genes played an important role in the development of fibers and ovules.

Interestingly, we also found that the expression patterns of *Ghrboh* genes in response to salinity was largely opposite to those obtained in response to drought stress. In other words, if the expression of a *Ghrboh* gene was up-regulated under salinity stress, then expression of the gene was down-regulated under drought stress. Specifically, expression of *Ghrboh2*/*10*/*11*/*14*/*20*/*22*/*23* was induced by salt but reduced by drought, whereas expression of *Ghrboh4*/*7* was induced by salt but reduced by drought. Not all genes exposed to salinity and drought stresses showed this ‘opposite trend’: *Ghrboh3* expression was up-regulated by both salinity and drought stresses, whereas *Ghrboh13* was significantly induced only by salinity (but not drought) at 12 h, and *Ghrboh12*/*21* was induced only by salinity stress (Additional file 1: Figure S4). The molecular mechanism of this interesting phenomenon needs to be studied further, a topic that we plan to report on in greater detail in the future.

These results suggested that the genes described above may be important for stress responses and/or developmental processes and will be useful in cloning candidate genes for functional analysis of their role in stress response and fiber development.

### The putative regulation mechanisms of *Ghrboh* gene expression

As evident from a number of studies, NOX-dependent ROS production of plant is associated with numerous stress-, morphogenesis- and development-related signaling pathways, such as phytohormone signaling pathways [12], although how the ROS flux mediated by the *Ghrboh* gene family is deciphered downstream to achieve a specific response has yet to be elucidated. In the current study, the prediction of transcription-related components, including *cis*-elements and post-transcriptional regulation mediated by upland cotton miRNAs, may provide an insight into the putative regulatory mechanisms underlying *Ghrboh* gene expression and their functional multiplicity.

In the *Ghrboh* promoter regions, we found a number of stress-response elements, such as ARE, Box-W1, HSE, LTR, WUN-motif and TC-rich repeats, which are responsive to biotic and abiotic stresses (Fig. 6 and Additional file 1: Table S4). We also found several phytohormone regulatory elements in the *Ghrboh* promoters, which indicated that the *Ghrboh* gene family probably participates in phytohormone-signaling pathways. Specifically, we noted the ABRE, TGA/AuxRR-core, ERE and GAREs elements, which were associated with ABA, ethylene, gibberellin and auxin responses, respectively (Fig. 6 and Additional file 1: Table S4).

It is reported that ABA accumulates under stress conditions and plays an important role in the stress response and tolerance of plants, which may coordinate the ROS signaling route [51]. Several evidences show that ABA induces ROS accumulation in the apoplast, which is dependent on *Rboh* genes and plays an important role in ABA signaling [52]. For instance, in *Arabidopsis*, 2 *Rboh* genes (*RbohD* and *F*) of 10 functioning *Rboh* genes (*RbohA*-*H*) had been shown to be involved in the ABA signaling [53]. In this study, the *cis*-acting element, ABA-responsive element (ABRE), was found in 15 *Ghrboh* genes promoter region (Fig. 6 and Additional file 1: Table S4). Based on the results of qPCR, we found that the vast majority of the 15 *Ghrboh* genes had significantly different expression patterns under drought and/or salt stress (Additional file 1: Figure S4). In addition to the ABA, phytohormones, such as auxin [54, 55], ethylene [56, 57] and gibberellin [58] are known to play important roles in cotton fiber development. The development of cotton fiber includes four overlapping stages, which are defined based on the number of dpa: initiation (− 5 to 5 dpa), elongation (2 to 30 dpa; the most active elongation period is 5 to 20 dpa), secondary cell wall accumulation (20 to 50 dpa; the rapid accumulation period is 25 to 40 dpa) and maturation (45 to 60 dpa) [59–61]. Previous studies had revealed that auxin accumulates in the ovule epidermis and fiber cells from − 5 to 10 dpa [55], and a substantial amount of ethylene and gibberellin were synthesized in the elongating fiber cells, with the biosynthesis of ethylene and gibberellin being two of the most significantly upregulated biochemical pathways during cotton fiber elongation [56, 62]. In the current study, based on the results of qPCR, we analyzed the expression patterns of those *Ghrboh* genes that carried the phytohormone-responsive elements in the promoters in both fibers and ovules during cotton fiber development, and found that the vast majority of them had significantly different expression patterns from one another at the corresponding time points of phytohormone accumulation during cotton fiber development. For instance, among the genes associated with auxin response, *Ghrboh3* showed significantly up-regulated expression at 10 dpa in the fiber and at − 1 and 0 dpa in the ovule, whereas *Ghrboh13* and *Ghrboh14* showed significantly up-regulated expression at 5 dpa in the fiber and from 5 to 10 dpa in the ovule (Additional file 1: Figure S2 and Additional file 1: Figure S3). Among the genes associated with ethylene response, *Ghrboh5* showed significantly down-regulated expression in the fiber during the most active fiber elongation period, from 5 to 20 dpa, whereas *Ghrboh9* showed significantly up-regulated expression from 5 to 10 dpa in the fiber, *Ghrboh23* showed significantly up-regulated expression at 5 and 20 dpa in the fiber and*Ghrboh10*/*24*/*25* all exhibited significantly up-regulated expression at 5 dpa in the fiber (Additional file 1: Figure S3). Among the genes associated with gibberellin response, *Ghrboh6* showed significantly down-regulated expression from 5 to 20 dpa in fiber, *Ghrboh7*/*22*/*23* showed significantly up-regulated expression at 5 and 20 dpa in the fiber and *Ghrboh8*/*12* showed significantly up-regulated expression from 10 to 20 dpa in the fiber (Additional file 1: Figure S3). These results suggested that these genes are probably responsive to phytohormones, and the *Ghrboh* gene family might be regulated by the *cis*-elements associated with phytohormone signaling during cotton fiber development and stress responses.

We also predicted miRNA-mediated post-transcriptional regulation of *Ghrboh*s and identified some putative target sites of upland cotton miRNAs. These miRNAs were divided into a conserved group (e.g. ghr-miR414, ghr-miR482, ghr-miR2949 and ghr-miR3627) and a novel group (e.g. novel_mir_2473, Mar-F-3-m0087 and ghr-miR2673). Previous studies had indicated that these conserved and novel miRNAs were involved in some biological processes, including responses to environmental stresses and regulation of cell growth, development and metabolism in association with cotton fiber development [32–35, 63, 64]. For instance, ghr-miR2949, ghr-miR3627 and novel_mir_2473 have been proposed to be involved in cotton fiber development [33], whereas ghr-miR414 and Mar-F-3-m0087 might be associated with stress response and genetic male-sterility in upland cotton [34, 65], respectively. Our results will help point us in the appropriate direction for further experiments to determine the biological functions of these miRNAs and their targets in upland cotton.

Plants respond to environmental stress and regulate growth and development in multiple ways and have evolved mechanisms to increase their tolerance to abiotic stresses and to modulate relevant metabolism processes through interactive molecular and cellular changes. These mechanisms involve multiple systems, the foundation of which is a cooperative action of signal cascade transduction networks, involving multiple genes. However, evidence on the upstream regulation of *Ghrboh*s and the downstream factors regulated by *Ghrboh*s at different levels is lacking. For instance, one of the mechanisms that contributes to ROS-induced pathogen tolerance is the activation of many enzymatic and nonenzymatic antioxidants, such as glutathione-S-transferases (GSTs), ascorbate peroxidases (APXs), superoxide dismutases (SODs), catalases (CATs), glutathione and ascorbic acid [66]. But the relationships between the innate plant immune system and the activation of antioxidants, as well as non-coding RNA-mediated stress tolerance in plant, needs further investigation.

## Conclusions

We identified 26 putative *rboh* genes distributed over 18 of the 26 chromosomes or scaffolds in the upland cotton genome. During the evolutionary process, WGD or polyploidy events and segmental duplications contributed to the expansion of the *Ghrboh* gene family. The expression patterns of the *Ghrboh* gene family were analyzed using RNA-Seq and qPCR and showed different expression patterns in different tissues/organs, at different developmental stages and under different stresses, indicating diverse functions in growth, development and stress response of cotton. The promoter sequence analysis revealed that there were many *cis*-acting elements associated with phytohormone and stress response, but different members harbored distinct types and numbers, which suggested that individual members of the *Ghrboh* gene family might be differentially regulated at the transcriptional level. Moreover, we also predicted and analyzed the miRNA-mediated post-transcriptional regulation of the gene family in this species. Taking all these results into account, we hypothesized that the *Ghrboh* gene family, which might be regulated by *cis*-elements and miRNAs at different levels, played roles in cotton development and stress tolerance through modulating NOX-dependent ROS induction. Collectively, our study provides a comprehensive analysis of and novel insights into the expression, regulation, and evolution of the *Ghrboh* gene family, and helps lay the foundation for further cloning and functional verification of the *Ghrboh* genes by reverse genetics research. Additionally, these results may increase our understanding of the molecular basis of many important traits in agronomic upland cotton, such as fiber development, pathogen resistance, and tolerance to abiotic stresses.

## Methods

### Identification of *Rboh* genes

The upland cotton genome files (*G. hirsutum*, NAU) were downloaded from the Cotton Functional Genomics Database (CottonFGD) (https://cottonfgd.org/) [67]. To identify the *rboh* genes in upland cotton, the BLAST algorithm for Proteins (BLASTP) [68] was performed using the full-length protein sequences coded by ten *rboh* genes from *A. thaliana* (Locus ID see Additional file 1: Table S3) and the Hidden Markov Model (HMM) profile of the NADPH_Ox (PF08414), EF-hand (PF00036), Ferric reductase NAD binding domain (PF08030) and FAD-binding domain (PF08022) obtained from Pfam (http://pfam.xfam.org/) [69] as the queries. InterProScan (version 4.8) [70] was further used to confirm the inclusion of the conserved domain of rboh in each candidate sequence using the Pfam database (http://pfam.xfam.org/). The *rboh* genes of the other 20 plant genomes obtained from the JGI database (http://www.phytozome.net) [71] and CottonGen (https://www.cottongen.org) [72] (Additional file 1: Figure S1) were identified using methods similar to those described above. The details of upland cotton *rboh* (*Ghrboh*) genes, including locus ID, genomic position, gene length and open reading frames length, were collected from the *G. hirsutum* genome and annotation files.

### Sequence and functional annotation analysis

The graphical visualization of *Ghrboh* genes exons-intron structures gathered from the GFF3 file of the upland cotton genome was performed by the Gene Structure Display Server (GSDS) (http://gsds.cbi.pku.edu.cn/) [73]. The ProtParam tool was used to calculate the physico-chemical characteristics of Ghrboh proteins (http://www.expasy.org/tools/protparam.html), including the number of amino acids, molecular weight, instability index and theoretical isoelectric point. Predictions of subcellular localizations of Ghrboh proteins were performed with CELLO v.2.5 (http://cello.life.nctu.edu.tw/) [74]. The conserved domains of all the protein sequences coded by candidate *Ghrbohs* were predicted with the Simple Modular Architecture Research Tool (SMART) (http://smart.embl-heidelberg.de/) [75]. The IBS software (http://ibs.biocuckoo.org/) [76], called illustrator for biological sequences, was used for preparing the Ghrboh protein functional domain graphs. Prediction of transmembrane helices in predicted upland cotton rboh proteins were performed with the TMHMM Server v. 2.0 (http://www.cbs.dtu.dk/services/TMHMM/).

### Synteny and collinearity analysis

The chromosomal location of *rboh* genes was drafted from top to bottom on upland cotton chromosomes according to gene positions in the genome annotation by Circos-0.69 (http://circos.ca/) [77]. A synteny analysis was conducted locally using a method similar to that developed for the Plant Genome Duplication Database (http://chibba.pgml.uga.edu/duplication/) [78]. We used BLAST+ version 2.6.0 [68] for the pairwise comparison of the filtered rboh protein sets of *G. hirsutum*, *G. raimondii* and *G. arboreum*. Then, MCscanX [79] was employed to identify homologous regions, and syntenic blocks and duplicate gene classifications were evaluated using Circos-0.69. Default parameters were used in all the steps.

### Phylogenetic and evolutionary analysis

The sequence data used in this study were collected from the National Center for Biotechnology Information (NCBI) (http://www.ncbi.nlm.nih.gov/protein/) and the JGI database (http://www.phytozome.net) [71]. The full-length coding sequences of the plant *rboh* genes were aligned by the ClustalW program with default parameters [80]. MEGA6.0 software was used to construct the phylogenetic trees with a bootstrap analysis of 1000 replicates and the neighbor-joining (NJ) method [81]. In addition, to further estimate *Ghrboh* genes duplication events, the non-synonymous (*Ka*) and synonymous (*Ks*) substitution rates of evolution were calculated using add_ka_and_ks_to_collinearity, the downstream analysis program of the MCScanX package [79]. To estimate the evolutionary duplication time of duplicated genes, *Ks* values were translated into duplication time in millions of years based on a rate of one substitution per synonymous site per year. The duplication events time (*t)* was calculated from the equation “*t* = *Ks*/2*r*”, where “*r*” was the neutral substitution rate. A neutral substitution rate of 2.6 × 10^− 9^ was used in the current study [31].

### Digital expression profiling analysis

Expression value (FPKMs) of *Ghrbohs* was obtained from the websites at CottonFGD (https://cottonfgd.org/) [67] and ccNET (http://structuralbiology.cau.edu.cn/gossypium/) [82]. We determined the expression differences of *Ghrbohs* in different tissues/organs (root, stem, leaf, petal, torus, stamen, pistil and calycle), at different developmental processes of ovules (− 3, − 1, 0, 1, 3, 5, 10, 20, 25 and 35 dpa) and fibers (5, 10, 20 and 25 dpa), and under different stress treatments (hot, cold, PEG and salt treatments) with log_2_(fold-change of *Ghrbohs* FPKMs) ≥1 and *p*-value < 0.05 (Fisher’s Exact Test).

### Plant materials and stress treatments

The *G. hirsutum* cv. SF06 plants were used in this research and were cultivated in a trial field from April to September under standard conditions in Tai’an, the experimental station of Shandong Agricultural University. Flowers were tagged on the day of anthesis, and cotton bolls were harvested at 0, 3, 5, 10, 20 and 25 days post-anthesis (dpa); the bolls at − 1 dpa were harvested based on the characteristics of cotton budding. We excised ovules from the bolls, and scraped fibers from the ovules at 5, 10, 20 and 25 dpa. All ovules and fibers were frozen in liquid nitrogen and stored at − 80 °C until total RNA was extracted. For the stress treatments, the seeds of upland cotton were sown in a soil mix [peat moss:perlite, 2:1 (v/v)] in plastic pots and were placed in plant growth chambers under the following conditions: 28 °C/21 °C day/night temperature, 16/8 h light/dark photoperiod, 3300 lx light intensity and a relative humidity of 70%. And, the uniform-sized plantlets were cultivated in Hoagland’s solution (pH = 5.6 and changing every 3 d) after the expansion of the first true leaf. Approximately one week later, the plantlets were treated, with the nutrient solution supplemented with 250 mM NaCl for the salt treatment, or 20% (v/v) polyethylene glycol (PEG) 6000 for the drought treatment. The leaves of treated plantlets were harvested after 0, 1, 3, 6 and 12 h stress treatment. All the harvested samples were frozen in liquid nitrogen and stored at − 80 °C until total RNA was extracted. Three independent biological replicates were performed for each treatment.

### Quantitative polymerase chain reaction (qPCR)

Total RNA from these samples was isolated using RNAprep Pure Plant Kit (Polysaccharides & Polyphenolics-rich, DP441) (TIANGEN, Beijing, China). The quality and concentrations of the isolated RNA samples were determined by 1.5% agarose gel electrophoresis and a NanoDrop 2000 Spectrophotometer (Thermo Fisher Scientific, Wilmington, DE, USA), respectively. Reverse transcription PCR was carried out using HiScript® II Q RT SuperMix for qPCR with gDNA wiper (R223) (Vazyme, Nanjing, China). Transcript levels were determined using a QuantStudio™ 6 Flex Real-Time PCR System (Applied Biosystems™, Carlsbad, CA, USA) and ChamQTM Universal SYBR® qPCR Master Mix (Q711) (Vazyme), with three technical replicates for each biological sample. PCR thermal cycling included an initial denaturation at 95 °C for 1 min, followed by 40 cycles at 95 °C for 10 s and 60 °C for 30 s in a reaction volume of 20 μl in 0.1 ml MicroAmp™ Fast Optical 96-Well Reaction Plate (4346907) (Applied Biosystems). Following the PCR, a melting curve analysis was performed. Cycle threshold was used for the relative quantification of the input target number. Relative fold difference represents the number of treated target gene transcript copies relative to the number of untreated gene transcript copies, and was calculated according to the 2^−ΔΔCT^ method [83]. To normalize the variance among samples, cotton ubiquitin 7 (*UBQ7*) was used as an endogenous control. Gene-specific primers used for qPCR were designed using Primer Premier 5.0 [84] and are listed in Additional file 1: Table S7. For statistical analysis, standardization of gene expression data was repeated at least three times with three biological replicates [85]. ANOVA (analysis of variance) was calculated using DPS (version 7.05) [86], and, if significant, the differences between samples were compared by LSD’s test (*p* < 0.05).

### Prediction of *Ghrbohs* regulatory elements

The genomic sequences at 1.5-kb upstream of the translation start site (TSS) of each *Ghrboh* gene were extracted from the genome files of *G. hirsutum* TM-1. The PlantCARE serve was used to predict the transcriptional response elements of *Ghrboh* gene promoters (http://bioinformatics.psb.ugent.be/webtools/plantcare/html/) [87]. We obtained cotton miRNA sequences from miRBase (http://www.mirbase.org/) [88], the Plant MicroRNA database (http://bioinformatics.cau.edu.cn/PMRD/) [89], the Cotton EST database (http://www.ncbi.nlm.nih.gov/nucest) and published articles. *Ghrboh* genes targeted by miRNAs were predicted by searching coding sequences (CDS) regions for sequences complementary to the cotton miRNAs, using the psRNATarget server with default parameters, except for maximum expectation (E) = 3.0 and maximum unpaired energy (UPE) = 20.0 (http://plantgrn.noble.org/psRNATarget/home) [90].

## Supplementary information

**Additional file 1: Table S1.** Orthologous *rboh* gene pairs of *G. hirsutum*, *G. arboreum*, and *G. raimondii*. **Table S2.** The non-synonymous (*Ka*) and synonymous (*Ks*) substitution and estimated age of the duplication events for *Ghrboh* paralogous genes. **Table S3.** Gene numbers of *rboh* gene family in 17 plant genomes. **Table S4.** List of identified *cis*-elements in the putative promoter region of 26 *Ghrboh* genes using PlantCARE web tool. **Table S5.** The details of predicted targeting regulatory relations between *Ghrbohs* and *G. hirsutum* miRNAs using psRNATarget web server. **Table S6.** The details of *Ghrbohs* expression difference in different tissues and/ororgans, at developmental processesof ovules and fibers, and under different stress treatments. **Table S7.** Gene-specific primers used for qPCR analysis of *Ghrboh* genes. **Figure S1.** Comparisons of *rboh* gene numbers across a wide range of organisms. **Figure S2.** Relative transcriptional expression levels of *Ghrbohs* in different developmental stages of upland cotton fiber by qPCR **Figure S3.** Relative transcriptional expression levels of *Ghrbohs* in different developmental stages of upland cotton ovule by qPCR. **Figure S4.** Relative transcriptional expression levels of *Ghrboh* under NaCl and PEG treatments by qPCR. **Figure S5.** Venn diagram analysis of *Ghrbohs* expression difference.

## Data Availability

All analysis results data generated during this study were included in this article and its Additional data repository.

## Abbreviations

aa: Amino acid
*Ac*: *Ananas comosus*
*Amt*: *Amborella trichopoda*
ARE: Anaerobic responsive element
*At*: *Arabidopsis thaliana*
*Bd*: *Brachypodium distachyon*
bp: Base pair
CDS: Coding sequence
*Cr*: *Chlamydomonas reinhardtii*
*Dc*: *Daucus carota*
DOA: Day of anthesis
DPA: Days post-anthesis
ERE: Ethylene-responsive element
FPKMs: Fragments per kilobase of transcript per million mapped reads
*Ga*: *Gossypium arboretum*
GAREs: GA-responsive elements
*Gh*: *Gossypium hirsutum*
*Gr*: *Gossypium raimondii*
HMM: Hidden Markov Model
HSE: Heat stress element
kb: Kilobase
LTR: Low-temperature responsiveness
*Ma*: *Musa acuminata*
*Md*: *Malus domestica*
miRNA: MicroRNA
*Mp*: *Micromonas pusilla*
*Mt*: *Medicago truncatula*
MW: Theoretical molecular weight of proteins
MYA: Million years ago
NADPH: Nicotinamide adenine dinucleotide phosphate
NOX: NADPH oxidase
nt: Nucleotide
*Ol*: *Ostreococcus lucimarinus*
ORF: Open reading frame
*Os*: *Oryza sativa*
PCD: Programmed cell death
PCR: Polymerase chain reaction
PCW: Primary cell wall
p*I*: Isoelectric point
*Pp*: *Physcomitrella patens*
qRT-PCR: Quantitative reverse-transcription polymerase chain reaction
rbohs: Respiratory burst oxidase homologs
ROS: Reactive oxygen species
*Sb*: *Sorghum bicolor*
SCW: Secondary cell wall
*Si*: *Setaria italica*
SOD: Superoxide dismutase
*Ta*: *Triticum aestivum*
*Tc*: *Theobroma cacao*
TF: Transcription factors
TFBS: Transcription factor binding sites
UBQ: Ubiquitin extension protein
UTR: Untranslated regions
*Vc*: *Volvox carteri*
*Vv*: *Vitis vinifera*
WGD: Whole genome duplication
WGT: Whole genome triplication
*Zm*: *Zea mays*
