# Supplementary material for: Comprehensive analysis of the Gossypium hirsutum L. respiratory burst oxidase homolog (Ghrboh) gene family
Source: BMC Genomics. 2020 Jan 29;21:91. doi: 10.1186/s12864-020-6503-6 (PMC6988335; doi:10.1186/s12864-020-6503-6)
Supplement: Supplementary file 1 — Additional file 1: Table S1. Orthologous rboh gene pairs of G. hirsutum, G. arboreum, and G. raimondii. Table S2. The non-synonymous (Ka) and synonymous (Ks) substitution and estimated age of the duplication events for Ghrboh paralogous genes. Table S3. Gene numbers of rboh gene family in 17 plant genomes. Table S4. List of identified cis-elements in the putative promoter region of 26 Ghrboh genes using PlantCARE web tool. Table S5. The details of predicted targeting regulatory relations between Ghrbohs and G. hirsutum miRNAs using psRNATarget web server. Table S6. The details of Ghrbohs expression difference in different tissues and/ororgans, at developmental processesof ovules and fibers, and under different stress treatments. Table S7. Gene-specific primers used for qPCR analysis of Ghrboh genes. Figure S1. Comparisons of rboh gene numbers across a wide range of organisms. Figure S2. Relative transcriptional expression levels of Ghrbohs in different developmental stages of upland cotton fiber by qPCR Figure S3. Relative transcriptional expression levels of Ghrbohs in different developmental stages of upland cotton ovule by qPCR. Figure S4. Relative transcriptional expression levels of Ghrboh under NaCl and PEG treatments by qPCR. Figure S5. Venn diagram analysis of Ghrbohs expression difference. [file 12864_2020_6503_MOESM1_ESM.pdf]

Additional files

Comprehensive Analysis of the *Gossypium hirsutum* L. Respiratory Burst Oxidase Homologs (*Ghrboh*) Gene Family

Wei Wang, Dongdong Chen, Dan Liu, Yingying Cheng, Xiaopei Zhang, Lirong Song, Mengjiao Hu, Jie Dong and Fafu Shen *

Contents

Additional file 1: Table S1. Orthologous *rboh* gene pairs of *G. hirsutum*, *G. arboreum*, and *G. raimondii*.

Additional file 2: Table S2. The non-synonymous (*Ka*) and synonymous (*Ks*) substitution and estimated age of the duplication events for *Ghrboh* paralogous genes.

Additional file 3: Table S3. Gene numbers of *rboh* gene family in 17 plant genomes.

Additional file 4: Table S4. List of identified *cis*-elements in the putative promoter region of 26 *Ghrboh* genes using PlantCARE web tool.

Additional file 5: Table S5. The details of predicted targeting regulatory relations between *Ghrbohs* and *G. hirsutum* miRNAs using psRNATarget web server.

Additional file 6: Table S6. The details of *Ghrbohs* expression difference in different tissues and/or organs, at developmental processes of ovules and fibers, and under different stress treatments.

Additional file 7: Table S7. Gene-specific primers used for qPCR analysis of *Ghrboh* genes.

(***All the tables were in the two separate Excel files.***)

Additional file 8: Figure S1. Comparisons of *rboh* gene numbers across a wide range of organisms.

Additional file 9: Figure S2. Relative transcriptional expression levels of *Ghrbohs* in different developmental stages of upland cotton fiber by qPCR.

Additional file 10: Figure S3. Relative transcriptional expression levels of *Ghrbohs* in different developmental stages of upland cotton ovule by qPCR.

Additional file 11: Figure S4. Relative transcriptional expression levels of *Ghrboh* under NaCl and PEG treatments by qPCR.

Additional file 12: Figure S5. Venn diagram analysis of *Ghrbohs* expression difference.

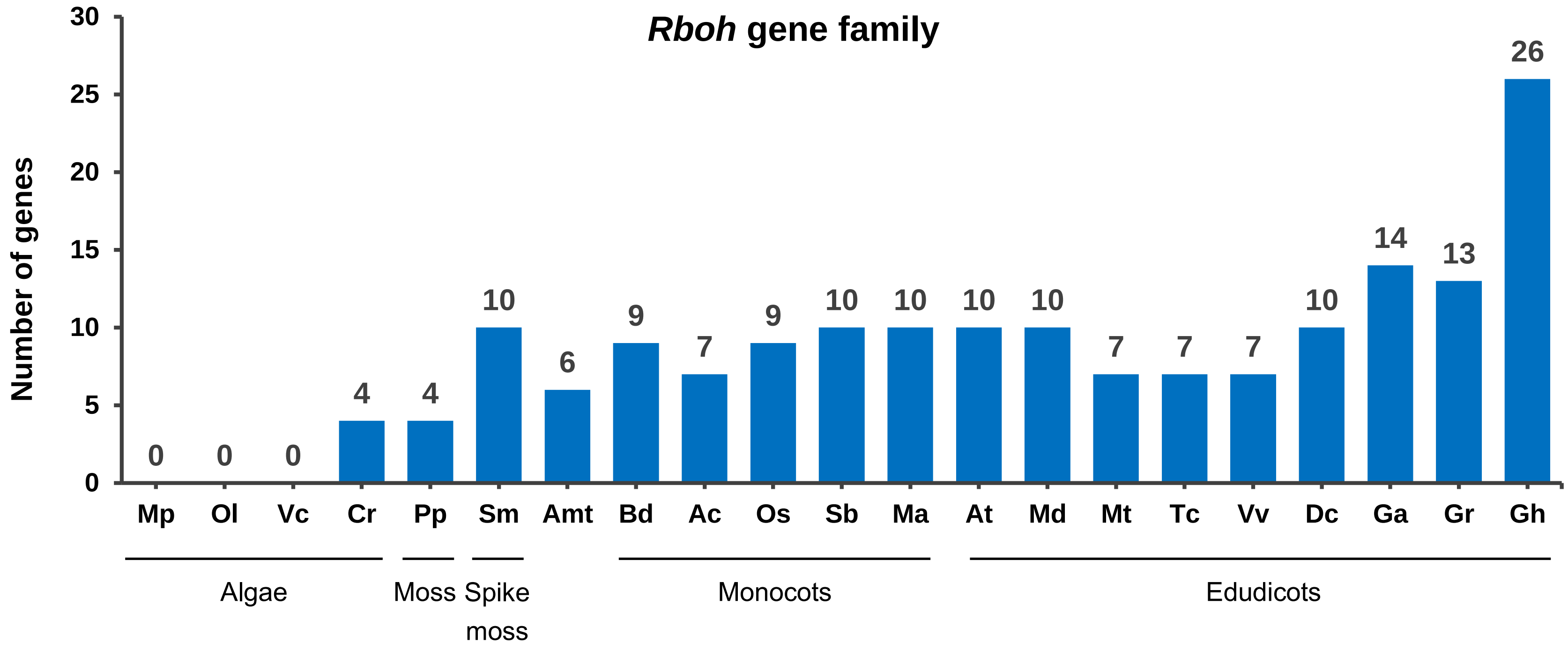

Additional file 8: Figure S1. Comparisons of *rboh* gene numbers across a wide range of organisms. **Mp**, *Micromonas pusilla*; **Ol**, *Ostreococcus lucimarinus*; **Vc**, *Volvox carteri*; **Cr**, *Chlamydomonas reinhardtii*; **Pp**, *Physcomitrella patens*; **Sm**, *Selaginella moellendorffii*; **Amt**, *Amborella trichopoda*; **Bd**, *Brachypodium distachyon*; **Ac**, *Ananas comosus*; **Os**, *Oryza sativa*; **Sb**, *Sorghum bicolor*; **Ma**, *Musa acuminata*; **At**, *Arabidopsis thaliana*; **Md**, *Malus domestica*; **Mt**, *Medicago truncatula*; **Tc**, *Theobroma cacao*; **Vv**, *Vitis vinifera*; **Dc**, *Daucus carota*; **Ga**, *Gossypium arboretum*; **Gr**, *Gossypium raimondii*; **Gh**, *Gossypium hirsutum*.

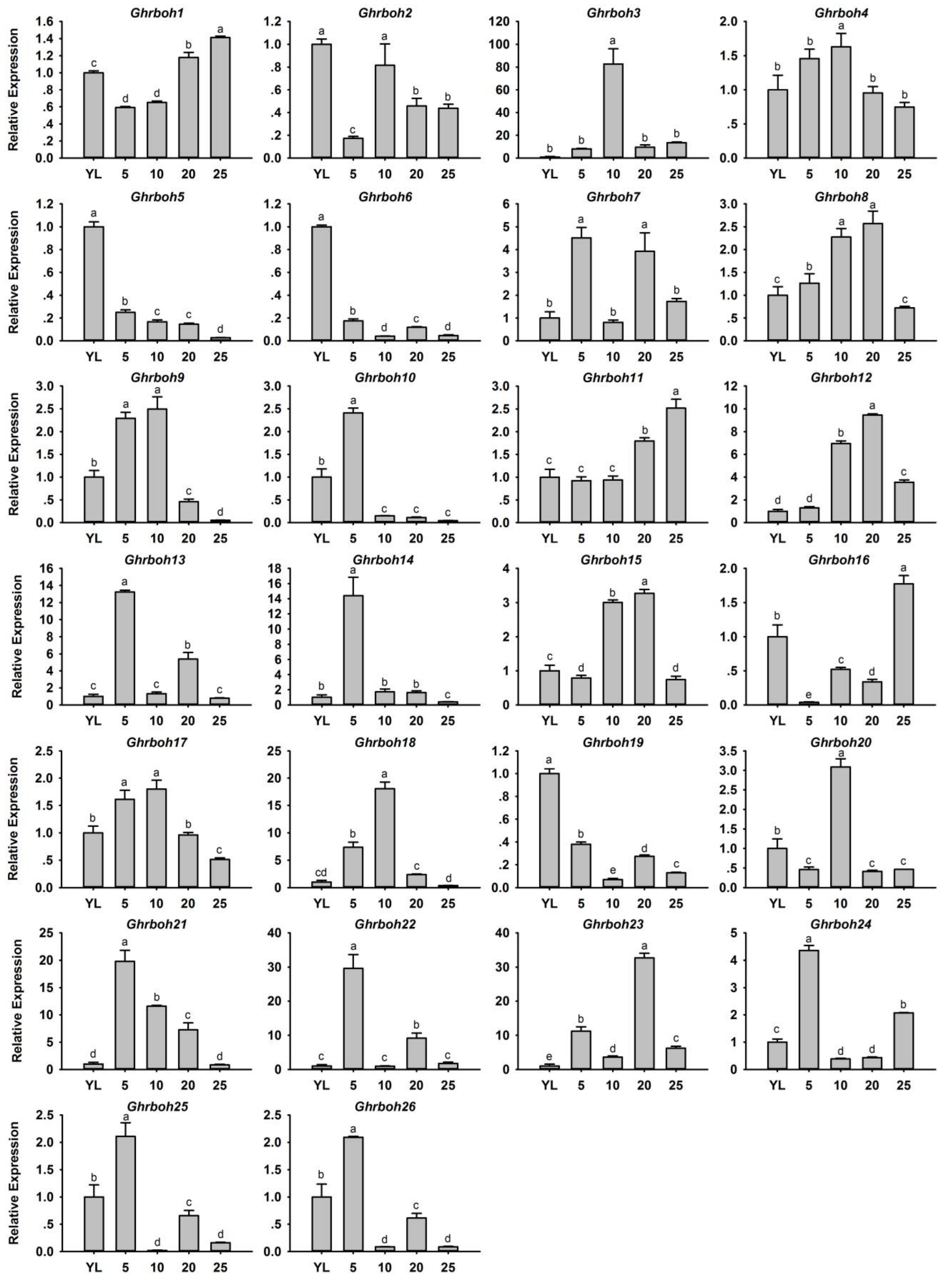

Additional file 9: Figure S2. Relative transcriptional expression levels of *Ghrbohs* in different developmental stages of upland cotton fiber by qPCR. YL, young leaves. Different developmental stages of fiber were shown on the *x*-axis, from left to right at the chart bottom: YL, 5dpa, 10dpa, 20dpa and 25dpa, and the relative expression levels on the *y*-axis. Transcript levels in YL were arbitrarily set to 1 and the levels in other tissues were given relative to this. Error bars represent standard deviations of mean value from three biological replicates. Means with a common letter are not significantly different at $p < 0.05$ according to LSD’s test.

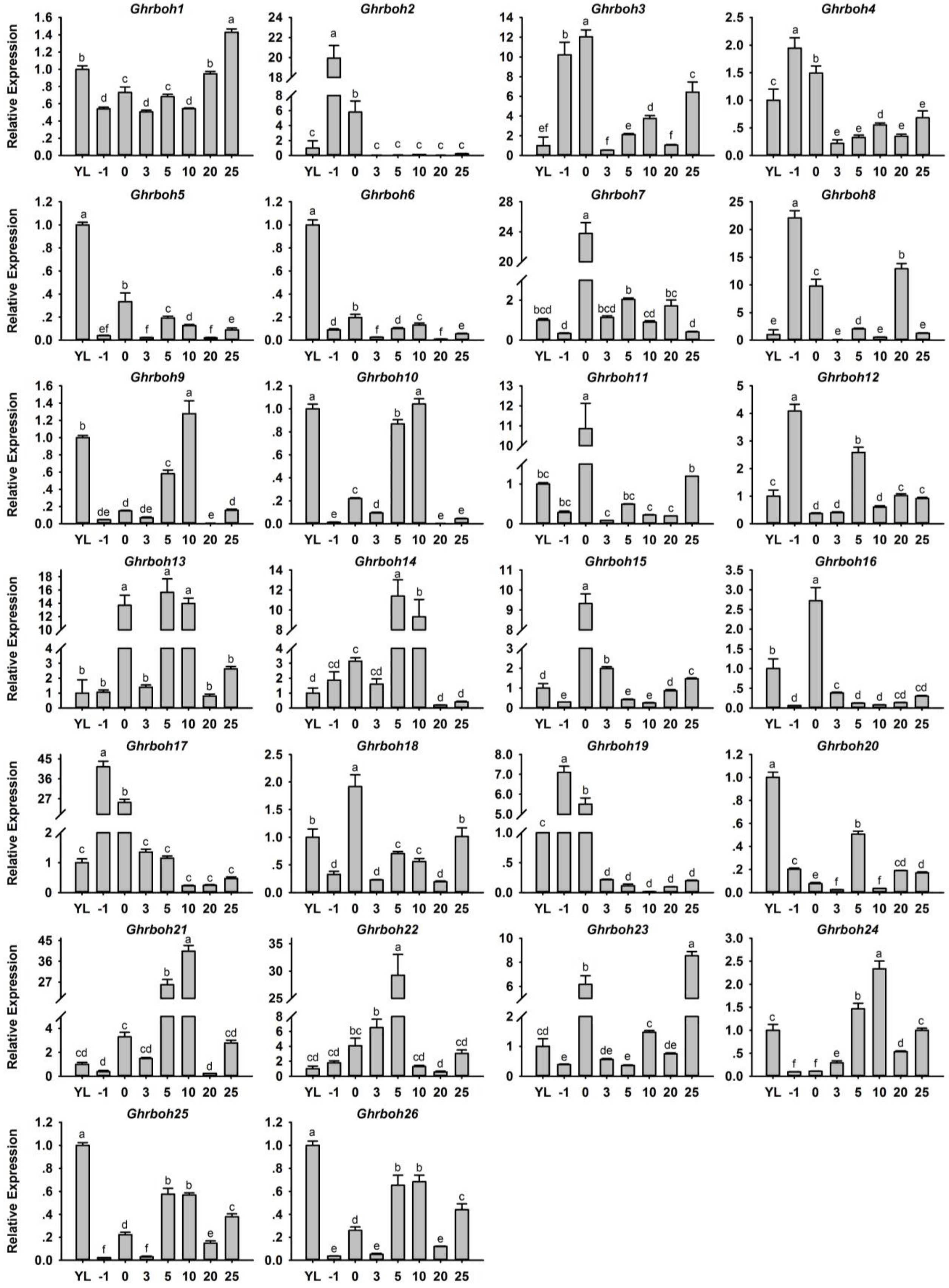

Additional file 10: Figure S3. Relative transcriptional expression levels of *Ghrbohs* in different developmental stages of upland cotton ovule by qPCR. YL, young leaves. Different developmental stages of ovule were shown on the *x*-axis, from left to right at the chart bottom: YL, -1dpa, 0dpa, 3dpa, 5dpa, 10dpa, 20dpa and 25dpa, and the relative expression levels on the *y*-axis. Transcript levels in YL were arbitrarily set to 1 and the levels in other tissues were given relative to this. Error bars represent standard deviations of mean value from three biological replicates. Means with a common letter are not significantly different at $p < 0.05$ according to LSD's test.

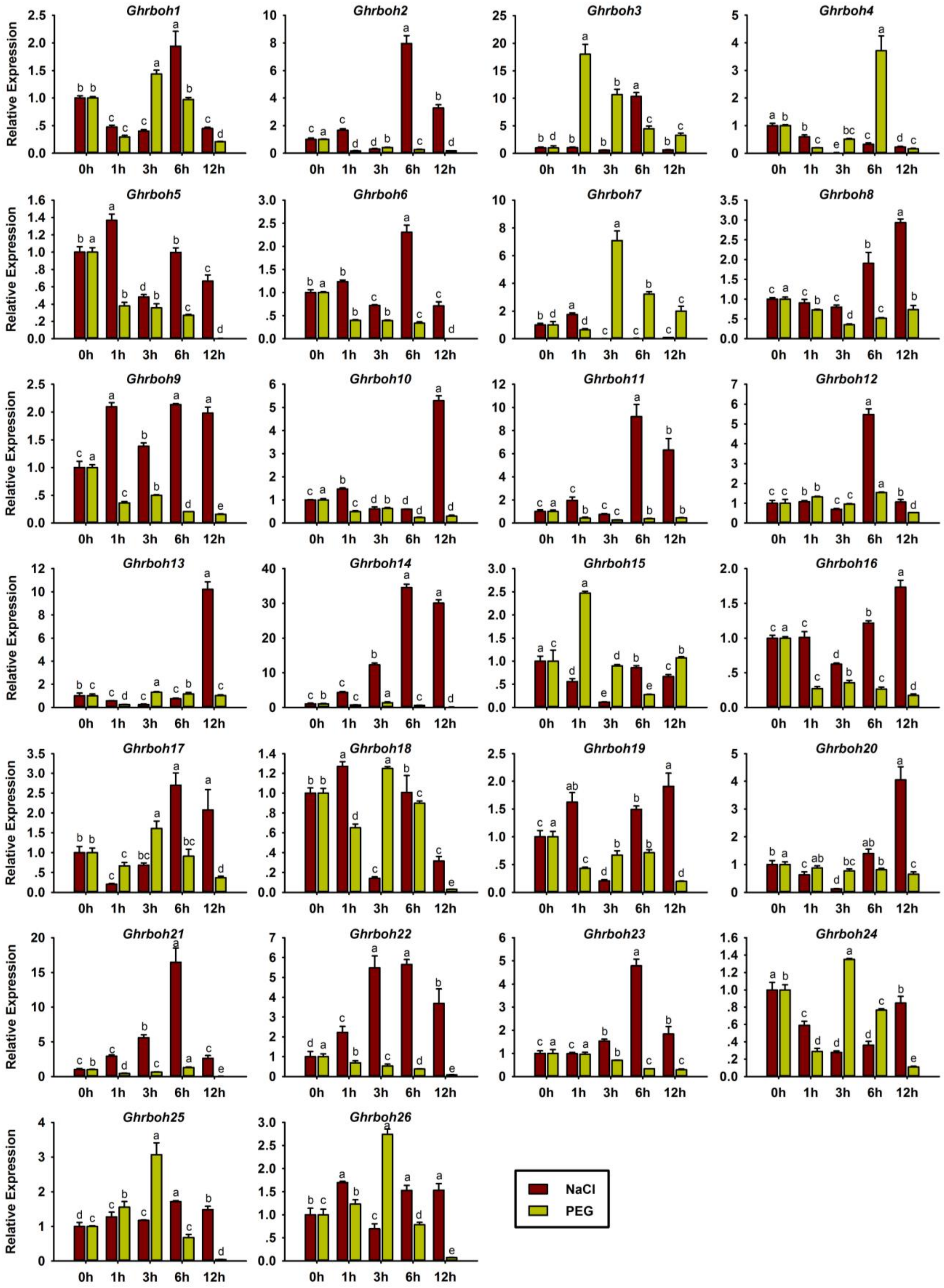

Additional file 11: Figure S4. Relative transcriptional expression levels of *Ghrbohs* under NaCl and PEG treatments by qPCR. Different stress treatment times were shown on the *x*-axis, and the relative expression levels on the *y*-axis. Error bars represent standard deviations of mean value from three biological replicates. Means with a common letter are not significantly different at $p < 0.05$ according to LSD's test.

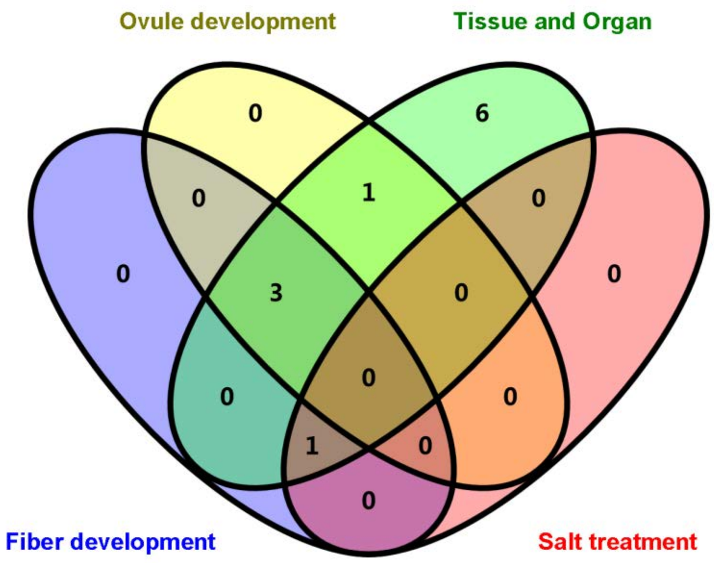

Additional file 12: Figure S5. Venn diagram analysis of *Ghrbohs* expression difference. The Venny 2.1 (http://bioinfogp.cnb.csic.es/tools/venny/index.html) was used to draw the Venn diagram.
